# Cloning and expression of *Burkholderia* polyyne biosynthetic gene clusters in *Paraburkholderia* hosts provides a strategy for biopesticide development

**DOI:** 10.1111/1751-7915.14106

**Published:** 2022-07-13

**Authors:** Yoana D. Petrova, Jinlian Zhao, Gordon Webster, Alex J. Mullins, Katherine Williams, Amal S. Alswat, Gregory L. Challis, Andy M. Bailey, Eshwar Mahenthiralingam

**Affiliations:** ^1^ School of Biosciences Cardiff University Cardiff UK; ^2^ Department of Chemistry University of Warwick Coventry UK; ^3^ School of Biological Sciences University of Bristol Bristol UK; ^4^ Warwick Integrative Synthetic Biology Centre University of Warwick Coventry UK; ^5^ Department of Biochemistry and Molecular Biology Biomedicine Discovery Institute Monash University Clayton Victoria Australia; ^6^ ARC Centre of Excellence for Innovations in Peptide and Protein Science Monash University Clayton Victoria Australia

## Abstract

*Burkholderia* have potential as biocontrol agents because they encode diverse biosynthetic gene clusters (BGCs) for a range of antimicrobial metabolites. Given the opportunistic pathogenicity associated with *Burkholderia* species, heterologous BGC expression within non‐pathogenic hosts is a strategy to construct safe biocontrol strains. We constructed a yeast‐adapted *Burkholderia‐Escherichia* shuttle vector (pMLBAD_yeast) with a yeast replication origin 2 μ and *URA3* selection marker and optimised it for cloning BGCs using the in vivo recombination ability of *Saccharomyces cerevisiae*. Two *Burkholderia* polyyne BGCs, cepacin (13 kb) and caryoynencin (11 kb), were PCR‐amplified as three overlapping fragments, cloned downstream of the pBAD arabinose promoter in pMLBAD_yeast and mobilised into *Burkholderia* and *Paraburkholderia* heterologous hosts. *Paraburkholderia phytofirmans* carrying the heterologous polyyne constructs displayed in vitro bioactivity against a variety of fungal and bacterial plant pathogens similar to the native polyyne producers. Thirteen *Paraburkholderia* strains with preferential growth at 30°C compared with 37°C were also identified, and four of these were amenable to genetic manipulation and heterologous expression of the caryoynencin construct. The cloning and successful heterologous expression of *Burkholderia* biosynthetic gene clusters within *Paraburkholderia* with restricted growth at 37°C opens avenues for engineering non‐pathogenic biocontrol strains.

## INTRODUCTION

*Burkholderia* is a phylogenetically diverse genus that thrive in a variety of environments, ranging from the rhizosphere to the cystic fibrosis lung (Eberl & Vandamme, 2016). Certain *Burkholderia* species such *Burkholderia glumae* are plant pathogens causing rot of rice grains (Jeong et al., 2003), whilst others like *Burkholderia ambifaria* form beneficial interactions with their plant hosts and protect them from fungal and bacterial pathogens (Mullins et al., 2019). Members of the *Burkholderia cepacia* complex (Bcc) found in the rhizosphere of wheat, maize and legumes have been shown to be useful as biocontrol agents, protecting the crops from damping‐off disease caused by oomycete and fungal pathogens including *Pythium* and *Fusarium* species (Bowers & Parke, 1993; Mao et al., 1998).

A key component of *Burkholderia's* biocontrol properties is biosynthetic gene clusters (BGC) encoding the production of diverse antimicrobial specialised metabolites, including alkaloids, polyenes, polyynes, macrolides, terpenes and quinolone derivatives (Kunakom & Eustaquio, 2019; Masschelein et al., 2017). Recently, by using comparative genomic approaches, the BGC encoding cepacin A biosynthetic enzymes was identified and demonstrated to be a key mediator in *B*. *ambifaria* supressing damping‐off disease caused by the oomycete *Globisporangium* (formerly *Pythium*) *ultimum* in *Pisum sativum (*Mullins et al., 2019 *)*. Cepacin was first isolated from ‘*Pseudomonas cepacia*’ (strain LMG 292043; now *Burkholderia diffusa*) and was shown to have good activity against *Staphylococcus aureus* but minimal activity against Gram‐negative organisms (Parker et al., 1984). Cepacin belongs to a group of compounds called polyynes, characterised by alternating single and triple carbon–carbon bonds. From bioinformatic analysis, the core *B*. *ambifaria* cepacin A BGC is approximately 13 kb and consists of 13 biosynthetic genes organised in a single operon with *luxRI* regulatory genes located upstream (Mullins et al., 2019). Another characterised *Burkholderia* polyyne is caryoynencin that was first discovered in *Burkholderia caryophylli* (Kusumi et al., 1987), with further characterisation of the metabolite and its BGC in *Burkholderia gladioli* (Ross et al., 2014). Caryoynencin has activity against Gram‐positive and Gram‐negative bacteria (Kusumi et al., 1987) and fungi (Florez et al., 2017).

Fungal and bacterial plant pathogens lead to major crop and economic losses, and there is an urgent need to develop new pesticides for use in agriculture (Savary et al., 2012). Cepacin and caryoynencin are potent antimicrobial molecules, but are unstable and challenging to purify (Mullins et al., 2021; Ross et al., 2014), making them difficult for a development into a direct‐application commercial product. However, a proven way to exploit the beneficial properties of polyynes has been to employ the producer strains directly as crop seed coats, enabling them to act as biopesticides (Mullins et al., 2019). Products containing live *Burkholderia* spp. were registered with the US Environmental Protection Agency (EPA) under the trade names Deny®, Blue Circle®, Intercept® (Parke & Gurian‐Sherman, 2001). However, concerns over the opportunistic pathogenicity of the Bcc species in cystic fibrosis (CF) and immunocompromised patients, coupled with an inability to distinguish between pathogenic and environmental strains, led to US Environmental Protection Agency (EPA) placing a moratorium on *Burkholderia*‐based biopesticides (Parke & Gurian‐Sherman, 2001). Consequently, given the concerns about safety (Parke & Gurian‐Sherman, 2001), the exploitation of *Burkholderia* as biocontrol agents has been limited in the last 20 years (Mullins et al., 2019).

Recently, advances in genomic‐based taxonomy have led to a split in the *Burkholderia* genus and the subsequent reclassification of a distinct clade of environmentally prevalent taxa into the new genus, *Paraburkholderia* (Sawana et al., 2014). Species such as *Paraburkholderia phytofirmans* has been particularly studied for their plant‐protective and growth‐promoting properties (Sessitsch et al., 2005). Isolated from onion roots (Frommel et al., 1991), *P*. *phytofirmans* strain PsJN (LMG 22146^T^) has restricted growth at 37°C (Sessitsch et al., 2005), making it unlikely to act as a human pathogen. Engineering non‐pathogenic *Paraburkholderia* strains to express biosynthetic gene clusters would be a way forward to improve the safety of future biocontrol preparations.

A limited number of plasmid vectors have been used for heterologous expression of genes in *Burkholderia*. The pMLBAD arabinose‐inducible system, capable of shuttling from *Escherichia coli* to *Burkholderia* has been used in several studies (Lefebre & Valvano, 2002; Masschelein et al., 2019). In addition to vectors, systems to clone and express biosynthetic gene clusters include the in vivo homologous recombination ability of *Saccharomyces cerevisiae* which offers multiple genetic engineering possibilities (Schimming et al., 2014). The yeast recombination strategy is a very efficient cloning method that enables the single‐step assembly of multiple fragments into a vector, requiring only 30 bases overlap between fragments (Oldenburg et al., 1997; Pahirulzaman et al., 2012).

Our objective was to evaluate whether complex BGCs from *Burkholderia* could be expressed in *Paraburkholderia* as a strategy for the development of biopesticides and biotechnological production platforms. This was met by developing an *E*. *coli‐Burkholderia‐S*. *cerevisiae* shuttle vector based on pMLBAD (Lefebre & Valvano, 2002) and employing the in vivo homologous recombination of *S*. *cerevisiae* for cloning *Burkholderia* polyyne BGCs. The suitability of the yeast‐adapted shuttle vector for gene pathway expression was initially evaluated and successfully demonstrated by placing a promoterless *luxCDABE* luminescent operon within it. Subsequently, the *B*. *ambifaria* cepacin A (from this point forward cepacin refers to cepacin A) and *B*. *gladioli* caryoynencin BGCs were cloned and investigated for metabolite production and bioactivity in different *Burkholderia* and *Paraburkholderia* strains. A panel of environmental *Paraburkholderia* isolates was also screened for defective growth at 37°C, and selected strains with restricted growth at these elevated infection‐associated temperatures were shown to be suitable for heterologous expression of polyynes. Overall, we describe a successful strategy for producing novel *Paraburkholderia* biocontrol and biotechnological strains that are capable of expressing high‐value *Burkholderia* specialised metabolite BGCs.

## EXPERIMENTAL PROCEDURES

### Microbial strains and molecular biology reagents

The bacterial and yeast strains used in the study are given in the Supplementary Materials (Table S1). In addition, a collection of 43 *Paraburkholderia* reference strains and novel isolates from the natural environment were screened as potential heterologous expression hosts (Table S2). Microbial strains used for polyyne susceptibility testing and the antagonism assay conditions are provided in Table S3. The plasmids (Table S4), PCR primers (Table S5) and PCR thermocycling conditions (Tables S6, S7 and S8) are also provided in the Supplementary Materials. Additional methods related to the strain collection, conjugal transfer of plasmids and their copy number, and metabolite analysis by high‐resolution mass spectrometry, and growth rate analysis at 30 vs. 37°C, are given in the Supplementary Information.

### Yeast adaptation of pMLBAD vector in *Saccharomyces cerevisiae*

The yeast fragment containing the replication origin 2 μ and the orotidine‐5′‐phosphate decarboxylase gene *URA3* (2867 bp) was amplified from pE‐YA plasmid (Pahirulzaman et al., 2012) (Table S4) using Q5® High‐Fidelity DNA Polymerase (NEB) with PCR thermal cycling conditions as per Table S6. The PCR primers, Yeast_fwd (forward primer) and Yeast_rev (reverse primer) (Table S5) were designed to incorporate 30 bp overlap with the pMLBAD vector backbone. The *E*. *coli*‐*Burkholderia* cloning plasmid pMLBAD was linearised with *Ase*I (NEB) and transformed into *S*. *cerevisiae* YPH500 alongside the yeast fragment PCR product, using the LiOAc yeast transformation method previously described (Pahirulzaman et al., 2012). The transformation mixture was plated on synthetic media (SM) containing 0.68% yeast nitrogen base without amino acids, 2.0% D‐glucose, 0.077% complete supplement mixture drop‐out URA (Formedium), 1.5% bacteriological agar 1 (Oxoid) and incubated at 30°C for 3–4 days until yeast colonies appeared. Plasmids were extracted from the yeast using Yeast Plasmid Miniprep (Zymo Research) and transformed into *E*. *coli* DH5α. The transformed *E*. *coli* were screened for the presence of the yeast fragment by colony PCR using Yeast_conf_fwd (forward primer) and Yeast_conf_rev (reverse primer), with PCR thermo‐cycling conditions as per Table S7.

### *luxCDABE* and polyyne pathway cloning using yeast‐adapted pMLBAD in *S*. *cerevisiae*

Prior to each transformation, pMLBAD_yeast plasmid was digested with *Hin*dIII and *Eco*RI (NEB). The polyyne pathways were PCR‐amplified in three overlapping fragments, whilst the *luxCDABE* operon in two overlapping fragments (see primers in Table S5), and overlap was 30 bp between the fragments and the vector backbone as appropriate. The cepacin BGC fragments were amplified from genomic DNA of *Burkholderia ambifaria* BCC0191, the caryoynencin fragments from *Burkholderia gladioli* BCC1697 and *luxCDABE* fragments from mini‐Tn5 *luxCDABE* plasmid (Winson et al., 1998). Q5® High‐Fidelity DNA polymerase was used to amplify the PCR fragments for each construct using the thermal cycling conditions (Table S6). The yeast transformation for each construct was performed as described above, with the yeast plasmids extracted and used to transform *E*. *coli* DH5α. Colony PCR with DreamTaq Polymerase (ThermoFisher Scientific) was used to confirm the presence of the correct constructs (Table S7). The construction of the plasmids was further confirmed by restriction digest and Sanger sequencing (Eurofins, UK) of a portion of the operon immediately downstream of Pbad promoter. Yeast homologous recombination was also used to replace the *araC*‐Pbad portion of the plasmid polyyne constructs with the native promoter of each polyyne cluster. Briefly, a kanamycin resistance cassette (including its transcriptional terminator) and the polyyne native promoter were PCR‐amplified with 30 bp homologous regions between them and with the plasmid backbone either side of the araC‐Pbad region. The homologous recombination in yeast yielded a polyyne construct with the native promoter directly upstream of the corresponding polyyne gene cluster and a kanamycin resistance cassette to allow for the selection of the correct constructs in *E*. *coli*.

### Luminescence assay for Pbad regulation characterisation

Luminescence assays were performed using a Tecan Infinite 200 PRO microplate plate reader for the bacteria harbouring the *luxCDABE* operon downstream of Pbad. Overnight cultures of strains containing pMLBAD_yeast_luxCDABE or pMLBAD_yeast_luxCDABE_rev were grown for 20 h at 30°C on a rocking platform (50 rpm) in minimal media containing 25 μg/ml trimethoprim; modified BSM‐G media without yeast extract or casamino acids was used for the *Burkholderia* and *Paraburkholderia* species. The cultures were then diluted to ~1 × 10^6^ cfu/ml in test media. Test media employed was minimal media with 25 μg/ml trimethoprim supplemented with either 0.2% (w/v) D‐glucose or L‐arabinose at a concentration range 0.05% to 0.8% (w/v). The cultures were grown for 24 h at 30°C in clear flat‐bottom 96‐microwell plates (200 μl per well; four technical replicates) on a rocking platform, and the optical density OD_600_ of the bacterial suspension was measured. The bacteria were then transferred to a white LUMITRAC flat‐bottom 96‐microplate, incubated in the dark for 10 min, followed by a 5 s orbital shake and measurement of the luminescence (relative light units; RLU). Each RLU measurement was divided by the corresponding OD_600_ to normalise for differences in cell densities. Response ratio for each strain under each test condition was calculated by dividing the normalised RLU value of the strain harbouring the pMLBAD_yeast_luxCDABE by the normalised RLU of the same strain containing pMLBAD_yeast_luxCDABE_rev.

### Plasmid stability in the absence of antibiotic selection

Overnight cultures of strains labelled with the pMLBAD_yeast_luxCDABE construct were grown for 20 h in 30°C on a rocking platform (50 rpm) in minimal media containing 25 μg/ml trimethoprim; modified BSM‐G media without yeast extract was used for the *Burkholderia* and *Paraburkholderia* species. The cultures were diluted to ~1 × 10^6^ cfu/ml in minimal media supplemented with 0.2% (w/v) L‐arabinose and 25 μg/ml trimethoprim, grown for 24 h at 30°C in clear flat‐bottom 96‐microwell plates (200 μl per well; four technical replicates) followed by OD_600_ and luminescence measures as described above to obtain initial measurements (day 0). The four technical replicates were then pooled, washed twice with sterile PBS to remove traces of antibiotic, diluted to ~1 × 10^6^ cfu/ml in antibiotic‐free minimal media containing 0.2% (w/v) arabinose and grown 24 h at 30°C in clear flat‐bottom 96‐microwell plates prior to obtaining the OD_600_ and luminescence measurements for day 1. The process was repeated for 3 passages in total. After each passage, the cultures were serially diluted and enumerated via drop counts on TSA plates with and without antibiotic (50 μg/ml trimethoprim) in order to calculate percentage of resistant colonies.

### Quantification of polyyne production using HPLC

Overnight cultures of strains were grown at 30°C on a rocking platform in tryptone soya broth (TSB), supplemented with 50 μg/ml trimethoprim. The cultures were adjusted to ~5 × 10^8^ cfu/ml and 7 × 20 μl streaks of the bacterial culture applied to solid test media containing 25 μg/ml trimethoprim. Following a 3‐day incubation at 22°C, the bacterial growth was removed with a sterile cell lifter, placed on pre‐weighed nitrocellulose filter, dried at 80°C for 24 h and the dry biomass weight recorded. 20 mm discs were excised from each agar plate and metabolites were extracted from the agar piece by incubation in 0.5 ml extraction solvent for 2 h with gentle shaking. Ethyl acetate (EtOAc) was used for the extraction of cepacin, whilst dichloromethane (DCM) was used for caryoynencin (Webster et al., 2020). HPLC analysis was conducted as previously described (Webster et al., 2020). Polyynes such as caryoynencin are known to be unstable (Ross et al., 2014). Key technical steps that enabled working with polyynes included: growing bacteria in the dark and keeping the incubation temperature low (22°C), extracting the compounds only from fresh ≤3 day growth plates, using a low water bath temperature (<30°C) to rotary evaporate extracts for LC–MS analysis, and not freeze‐drying extracts to concentrate or store them.

### Bioactivity overlay and contact antagonism assays with native and heterologous hosts

Test strains of native and heterologous polyyne hosts were tested for bioactivity against a range of susceptibility organisms using either an overlay (Mahenthiralingam et al., 2011; Mullins et al., 2019) or contact antagonism assay (Table S3). Overnight cultures of test strains grown at 30°C on a rocking platform in TSB, supplemented with 50 μg/ml trimethoprim were adjusted to ~5 × 10^8^ cfu/ml. The adapted overlay assay (Mahenthiralingam et al., 2011) involved inoculating 2 μl (~1 × 10^6^ cfu) of each test organism at the centre of a 90‐mm Petri dish and incubating at 22°C for 48 h. The bacteria were killed by exposing them to chloroform vapour for 3 min and overlaid with 15 ml half‐strength iso‐sensitest agar (Oxoid) seeded with ~1 × 10^6^ cfu/ml of susceptibility organism. The plates were incubated at the optimum incubation temperature and duration for each susceptibility organism (Table S3) and the diameter of the zones of inhibition measured in mm.

The contact antagonism assay was performed as described with slight modifications (Tenorio‐Salgado et al., 2013). Briefly, 6 mm diameter mycelial disc was excised from a 7‐day‐old potato dextrose agar (PDA) culture of oomycetes and filamentous fungi tested (see Table S3 for details of strains), placed in the centre of a 90‐mm Petri dish and incubated for 24 h at 22°C. The Petri dishes were the inoculated with 10 μl bacterial culture, adjusted to 5 × 10^8^ cfu/ml, in the form of four 15‐mm long streaks placed 30 mm away from the centre of the mycelial disc; a control plate without bacteria was used for each susceptibility organism used. Following a 6‐day incubation at 22°C, the bacterial antagonism was calculated percentage inhibition on mycelial growth as described (Tenorio‐Salgado et al., 2013). The assay for *Gl*. *ultimum* was slightly modified to account for faster growth of the organism—the bacterial streaks were inoculated to the Petri dish and incubated for 24 h at 22°C, following the addition of a mycelial disc from a 3‐day‐old PDA *Gl*. *ultimum* culture. The radial inhibition percentage was calculated following a further 48 h incubation.

## RESULTS

### Yeast‐adapting the *E*. *coli‐Burkholderia* pMLBAD vector

**FIGURE 1:**
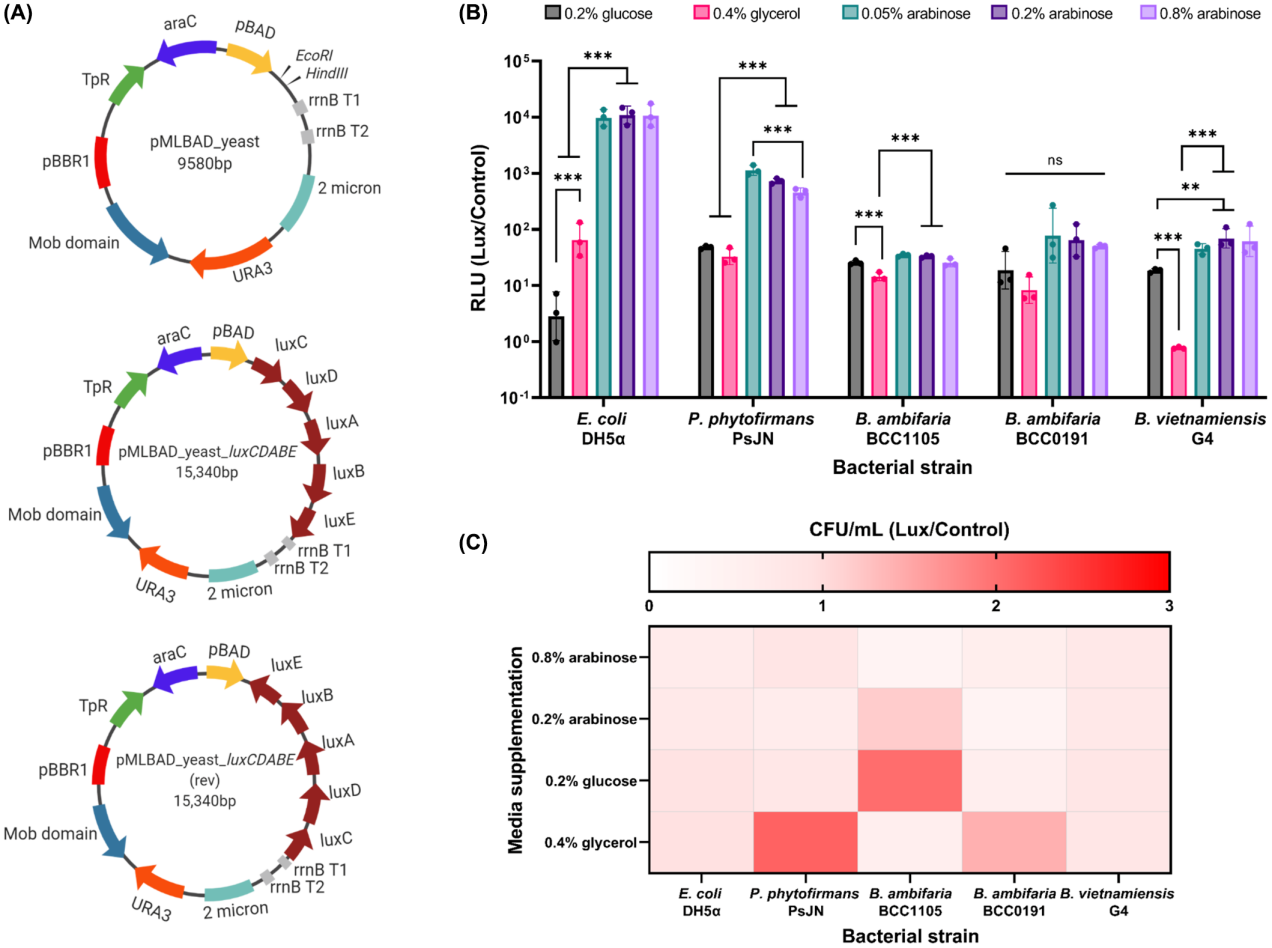
Cloning and expression of *luxCDABE* operon in yeast‐adapted pMLBAD. (A) Plasmid maps pMLBAD_yeast, pMLBAD_yeast_luxCDABE_rev, pMLBAD_yeast_luxCDABE (pBBR1_oriV = replication origin of the broad host range plasmid pBBR1 from *Bordetella bronchiseptica*; pBBR1_rep = replication protein for broad host range plasmid pBBR1 from *B*. *bronchiseptica*; TpR = trimethoprim resistance conferred by dihydrofolate reductase; araC = L‐arabinose regulatory protein; Pbad = promoter of the L‐arabinose operon in *E. coli*; rrnB T1 and T2 = transcription terminators of the *E. coli* *rrnB* gene; mob = mobilisation protein). (B) The bar chart shows the luminescence in relative light units (RLU) emitted by the bacteria harbouring the pMLBAD_yeast_luxCDABE plasmid divided by the RLU emitted by control (the bacteria containing pMLBAD_yeast_luxCDABE_rev). The luminescence was measured after 24 h growth in minimal media, supplemented with different concentrations of L‐arabinose or D‐glucose and was normalised by the optical density at 600 nm (OD_600_); basal levels of luminescence were measured after growth in glycerol. Statistical significance (***), (**) and (ns) corresponding *p* < 0.001, *p* < 0.01 and *p* > 0.05 was determined by one‐way ANOVA in GraphPad prism 9.0.2. The height of each bar represents the mean of three independent replicates, and the error bars show the SD (standard deviation). (C) Heatmap showing the viable count of the bacteria harbouring the pMLBAD_yeast_luxCDABE plasmid divided control (the bacteria containing pMLBAD_yeast_luxCDABE_rev.

The vector pMLBAD was selected as the basis for the transgene expression study due to its successful historical use in *Burkholderia* and *E*. *coli*, and its arabinose‐inducible expression (Lefebre & Valvano, 2002). The first step in design was to adapt the vector to allow yeast‐based recombination cloning (Pahirulzaman et al., 2012). The yeast 2 μ origin of replication was derived from an endogenous 2 μ plasmid, which is a high‐copy, stable and non‐selectable yeast plasmid (Ludwig & Bruschi, 1991). The *URA3* gene encodes orotidine‐5′‐phosphate decarboxylase, allowing the survival of a *URA3‐*deficient strain of *S*. *cerevisiae* in the absence of uracil (Boeke et al., 1987). These yeast markers were amplified from the yeast plasmid pE‐YA and cloned into pMLBAD to give the yeast‐adapted vector, pMLBAD_yeast (Figure 1A).

### Expression of *luxCDABE* pathway from yeast‐adapted pMLBAD vector

Having yeast‐adapted the pMLBAD vector, our next step was to show that this can express transgenes under arabinose‐based induction. The reporter system selected for this was the *Photorhabdus luminescens* luciferase operon *luxCDABE* (Winson et al., 1998), which consists of five genes, with *luxA* and *luxB* encoding a heterodimeric luciferase, whilst *luxC*, *luxD* and *luxE* encode the enzymes responsible for the biosynthesis of the luciferase substrate (Close et al., 2012). Using yeast recombination, the promoterless *luxCDABE* gene cluster was cloned in pMLBAD_yeast downstream of the Pbad promoter, in two fragments (*luxCD* and *luxABE*), with 30 bp overlap between them, to make pMLBAD_yeast_*luxCDABE* (Figure 1A). A construct containing the *luxCDABE* operon in reverse orientation to the Pbad promoter, pMLBAD_yeast_*luxCDABE_*rev (Figure 1A), was also assembled to act as a control for the background luminescence due to read through transcription. Detailed restriction digest maps for both vectors are given in the Supplementary Information (Figure S1A, B).

The Pbad promoter of pMLBAD has been shown to be activated by addition of 0.2% (w/v) arabinose and supressed by addition of 0.2% (w/v) glucose in both *E*. *coli* and *Burkholderia* (Guzman et al., 1995; Lefebre & Valvano, 2002). This regulation was assessed in heterologous hosts, *B*. *ambifaria*, *B*. *vietnamiensis* and *P*. *phytofirmans*, using the pMLBAD_yeast_*luxCDABE* construct as a positive expression control, prior to the more complex polyyne BGC cloning. The expression of the luciferase operon under the Pbad promoter was assayed by comparing the response ratios (see Methods) at different concentrations of L‐arabinose and in the presence of D‐glucose (Figure 1B, C). In *E*. *coli*, the basal level response ratio in the absence of L‐arabinose or D‐glucose, increases 100‐fold when arabinose is added at a concentration 0.05% (w/v) (Figure 1B). Further increasing the arabinose concentration to 0.8% did not yield significant increase in the response ratio in this host species (Figure 1B). Adding 0.2% (w/v) glucose significantly (*p* < 0.001; one‐way ANOVA; *F*_4,10_ = 9.692) suppresses the Pbad promoter in *E*. *coli* from the basal uninduced level, consistent with previous reports on Pbad promoter regulation in *E*. *coli* (Winson et al., 1998).

To our knowledge, there are no previous studies exploring the regulation of Pbad in *Burkholderia ambifaria* or *Paraburkholderia phytofirmans*. Pbad regulation performed in the species *B*. *cepacia* and *B*. *vietnamiensis* strains showed that addition of 0.2% (w/v) L‐arabinose leads to activation from baseline and addition of 0.2% (w/v) glucose to repression, measured by GFP fluorescence (Lefebre & Valvano, 2002). Our results demonstrate that addition of 0.05% (w/v) arabinose leads to an increased response ratio from basal level in *P*. *phytofirmans* PSJN, *B*. *vietnamiensis* G4, *B*. *ambifaria* BCC1105, but not *B*. *ambifaria* BCC0191 (Figure 1B). Increasing the arabinose concentration above this did not lead to significant increases in *lux* response ratio; in *P*. *phytofirmans* PsJN 0.05% L‐arabinose yielded greater response ratios than 0.8% arabinose (*p* < 0.001; one‐way ANOVA; *F*_4,10_ = 198.2). Interestingly, our results also suggest that the addition of 0.2% D‐glucose does not suppress the Pbad promoter below the basal level in the 3 *Burkholderia* species examined; in the case of *B*. *ambifaria* BCC1105 and *B*. *vietnamiensis* G4, the presence of glucose led to increases in response ratio (Figure 1B). However, an interesting result was the reduced light emission of *B*. *vietnamiensis* grown in the presence of 0.4% glycerol which demonstrated for this species there was either a significant lack of Pbad induction (compared with when either glucose or arabinose was present), or that an active repression of the promoter by glycerol was occurring (Figure 1B).

Since the addition of concentrations of L‐arabinose above 0.05% did not lead to any significant increase in the response ratio, we explored the possibility that the increased arabinose concentrations may have led to promoter induction, but the higher expression levels of the *luxCDABE* caused toxicity and cell death. However, the comparison of viable cell numbers for all the pMLBAD_yeast_*luxCDABE* constructs did not show this was occurring (Figure 1C).

### Cepacin and caryoynencin BGC cloning and heterologous expression

For the construction of pMLBAD_yeast_Pbadcep and pMLBAD_yeast_Pbadcay, the 13 kb cepacin gene cluster (Figure S2A) from *B*. *ambifaria* BCC0191 (Mullins et al., 2019) and the 11 kb caryoynencin gene cluster (Figure S2A) from *B*. *gladioli* BCC1697 (Jones et al., 2021) were PCR‐amplified and cloned into pMLBAD_yeast (Figure S2B). Cloning of the biosynthetic pathways was carried out using a design which incorporated 6–9 bases upstream of the first ATG codon of the operon and placed the promoterless gene cluster downstream of the Pbad promoter. The already optimised Shine Dalgarno of pMLBAD (Lefebre & Valvano, 2002) was used for both constructs. Multiple constructs containing the cepacin and caryoynencin BGC were obtained, and one from each experiment was evaluated for heterologous expression.

**FIGURE 2:**
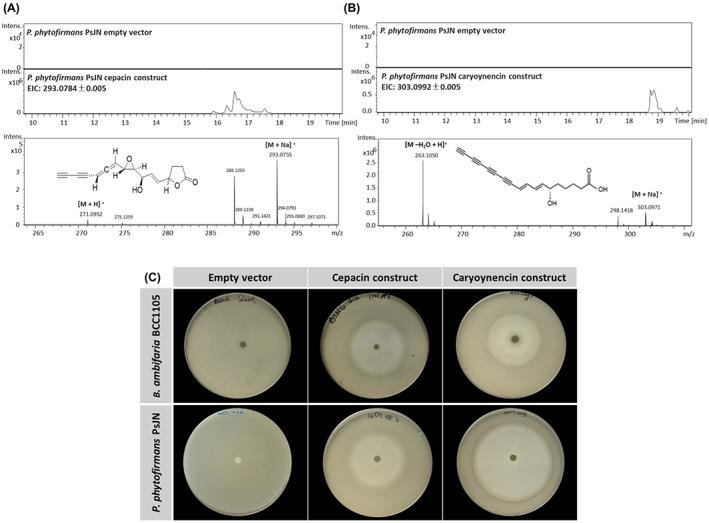
Heterologous expression of cepacin and caryoynencin under Pbad in *B. ambifaria* BCC1105 and *P. phytofirmans* PsJN. The extracted ion chromatograms (EIC) and high‐resolution LC–MS of the *P. phytofirmans* PsJN confirm the production of cepacin a (A) (EIC 15.8–17.0 min) and caryoynencin (B) (EIC 18.5–19.0 min) by the recombinant *P. phytofirmans* PsJN; no compound was detected in the empty vector control. The major peaks in the chromatograms correspond to cepacin a (A) and caryoynencin (B), respectively, and the mass spectra of these peaks is shown in the panel below. (C) The antagonistic activity against *S. aureus* of the recombinant *B. ambifaria* BCC1105 and *P. phytofirmans* PsJN, containing either a polyyne BGC under the control of Pbad or empty plasmid vector, determined using a classic overlay assay. The observed zones of clearance indicate activity against *S. aureus*.

Comparative LC–MS analyses confirmed that cepacin and caryoynencin are produced by the recombinant *P*. *phytofirmans* PsJN (Figure 2A, B) and *B*. *ambifaria* BCC1105 (Figures S3C and S4C) strains. The *B*. *ambifaria* BCC1105 and *P. phytofirmans* PsJN hosts containing the cloned cepacin and caryoynecin BGCs also gained new antagonistic activity against *S. aureus* (Figure 2C), a bacterium specifically susceptible to polyynes (Webster et al., 2020).

**FIGURE 3:**
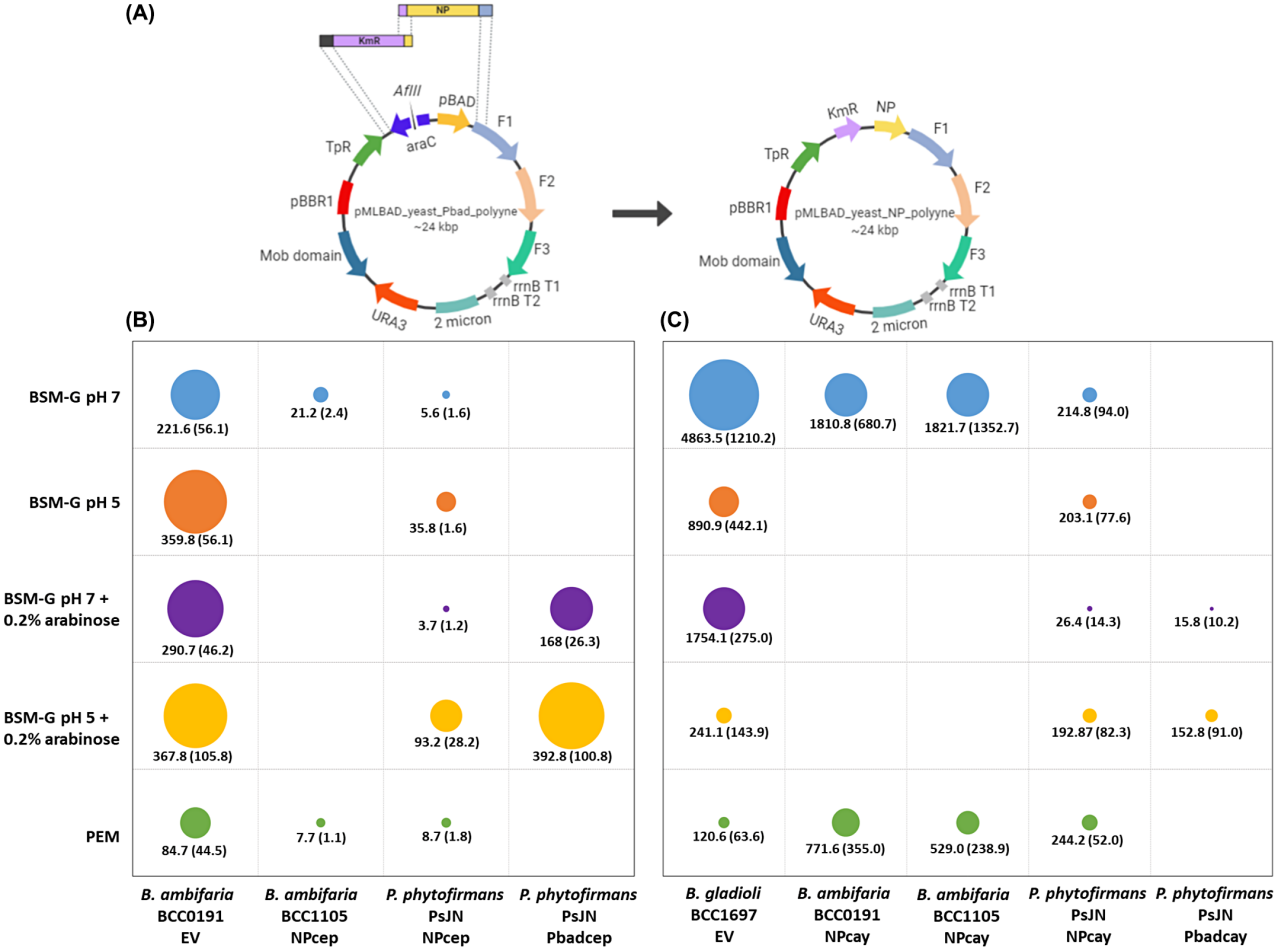
Swapping Pbad for native polyyne promoter and heterologous expression comparison. (A) Cloning strategy for swapping the BAD promoter of the pMLBAD_yeast_polyyne constructs for the native promoter of each BGC. F1, F2 and F3 represent the overlapping PCR fragments used to clone each respective polyyne pathway (see Table S5 for PCR primers). Yeast homologous recombination was used to replace the araC‐Pbad portion of the constructs with the native promoter (NP) fused to a kanamycin‐resistant cassette (KmR). The production of caryoynencin (B) and cepacin (C) by the native and recombinant hosts on different media types displayed in the bubble chart, with the size of each bubble representing the integrated HPLC peak area normalised by the dry biomass. The mean size of each bubble (*n* = 9) is displayed underneath with the standard deviation (*SD*) in brackets. The empty cells in the chart show the absence of polyyne detection.

Following successful expression of the cepacin and caryoynencin BGCs under the Pbad promoter in *Burkholderia* and *Paraburkholderia* host backgrounds, replacement of the arabinose promoter for the native promoter for each polyyne cluster was carried out as shown (Figure 3A). Exploring heterologous expression driven by the native promoter enabled further comparison of polyyne production on different growth media, including a biomimetic pea exudate medium (PEM), reflecting nutrients available during pea germination and therefore mimicking the biocontrol assay conditions previously described (Mullins et al., 2021). In addition, if polyyne expression occurred from the native promotors, it would facilitate future engineering approaches that do not need supplementation with L‐arabinose. Furthermore, understanding heterologous BGC expression from native promoters is helpful prior to moving forward with synthetic biology approaches using engineered regulatory elements (Li et al., 2017). Finally, arabinose may have acted as an alternative carbon source which is also known to influence *Burkholderia* BGC expression (Mahenthiralingam et al., 2011).

Since the polyynes are challenging to quantify due to their inherent instability (Mullins et al., 2021; Ross et al., 2014), a semi‐quantitative method of comparing HPLC peak areas was employed (Figure 3) (see Methods). The HPLC peak area of the native polyyne producer on a well‐characterised specialised metabolite induction growth medium, BSM‐G pH 7 (Mahenthiralingam et al., 2011; Mullins et al., 2019), was taken as a benchmark from which the induction level of all the other metabolite peak areas was evaluated (Figure 3B, C). Greater quantities of cepacin and caryoynencin were produced by the native producers, *B. ambifaria* BCC0191 (Figure 3B; EV) and *B. gladioli* BCC1697 (Figure 3C; EV), respectively, when grown on BSM‐G compared to PEM. The difference was particularly striking in *B. gladioli* BCC1697 with 28‐fold more caryoynencin produced on BSM‐G medium (Figure 3C; EV). In addition, growth at pH 5 vs. pH 7 altered the polyyne production, causing an increase in cepacin production by *B. ambifaria* BCC0191 (Figure 3B), but a decrease in caryoynencin for *B. gladioli* BCC1697 (Figure 3C). PEM also naturally reached a pH of 5.5, but the production of both polyynes was lower on this growth medium as noted above.

Polyyne production by the heterologous hosts was also dependent on the medium type. The production of caryoynencin by the heterologous hosts grown on BSM‐G was between 18‐fold (*P. phytofirmans* PsJN; Figure 3C, NPcay) and 3‐fold (*B. ambifaria* BCC1105; Figure 3C, NPcay) lower than the native producer *B. gladioli* BCC1697 (Figure 3C; EV). However, the normalised peak areas obtained for caryoynencin production on the biomimetic PEM were comparable between the native and heterologous hosts (Figure 3C). It was also interesting to observe that the caryoynencin BGC is expressed in *B. ambifaria* BCC0191, the native cepacin producer, and results in the recombinant strain producing both of the polyyne compounds. The production level of cepacin in *P. phytofirmans* PsJN and *B. ambifaria* BCC1105 when grown on BSM‐G was also lower than the native host *B. ambifaria* BCC0191 (Figure 3B; EV). The presence of L‐arabinose in the media also influenced the production of polyynes by the native hosts, leading to an increase in cepacin production by *B. ambifaria* BCC0191 (Figure 3B; EV) and a decrease in caryoynencin production by *B. gladioli* BCC1697 (Figure 3C; EV). This further supported the rationale to explore the use of native promoters in heterologous expression in work going forward.

### pMLBAD_yeast stability and copy number in heterologous hosts

Overall, the pMLBAD_yeast vector proved to be suitable for the heterologous of expression of polyyne BGCs under the control of both the arabinose‐inducible and native promoters. Next, we investigated the stability of the shuttle vector in the absence of antibiotic selection, using the *luxCDABE* construct which provided a direct readout of functional pathway expression efficacy as a reporter construct. Both light emission (Figure S5A) and the number of viable bacterial cells carrying the plasmid resistance marker (Figure S5B) diminished within 3 days for both the *Burkholderia* and *Paraburkholderia* hosts; in constrast the luciferase carrying plasmid remained stable in *E. coli* (Figure S5). Using quantitative PCRs targeting the plasmid vs. chromosome of each host (see Supplementary Methods), the copy number of the pMLBAD_yeast vector and recombinant polyyne pathway clones was found to vary between 2 and 19 copies per cell, dependent on the strain, host species and the specific construct. The copy number of all constructs in *P. phytofirmans* never exceeded 3, but in *B. ambifaria* the empty vector was at 18 copies per cell in strain BCC1105, with the cepacin construct reaching a copy number of 11 in the same host (Figure S5C).

### Bioactivity of native and heterologous polyyne‐producing hosts

**FIGURE 4:**
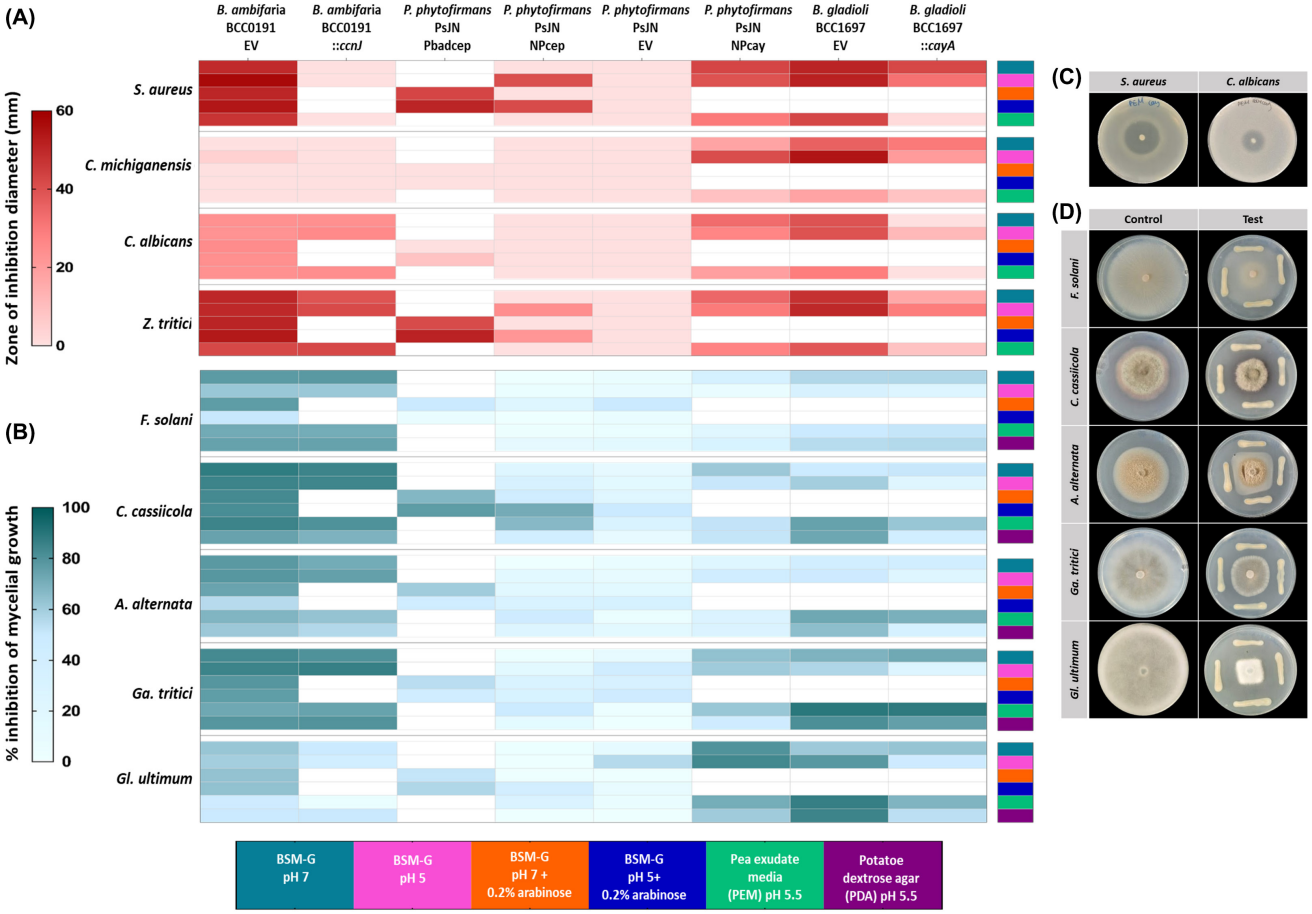
Heatmap of the bioactivity of native polyyne hosts, polyyne mutants and heterologous hosts. The heatmap shows the native polyyne producers *B. ambifaria* BCC0191 (cepacin) and *B. gladioli* BCC1697 (caryoynencin) with an empty vector (EV) pMLBAD_yeast; the polyyne inactivation mutants *B. ambifaria* BCC0191::ccnJ (cepacin inactivation) and *B. gladioli* BCC1697::cayA (caryoynencin inactivation); the recombinant *P. phytofirmans* PsJN producing either cepacin (NPcep and Pbadcep) or caryoynencin (NPcay and Pbadcay); and the empty vector control *P. phytofirmans* PsJN EV. The bioactivity of the strains was assessed on basal minimal media (BSM‐G) with/without 0.2% arabinose at pH 5 and pH 7, potato dextrose agar (PDA; pH 5.5) and pea exudate media (PEM; pH 5.5). White cells in the heatmap represent absence of measurement for that condition. (A) the bioactivity of the bacterial strains against two gram‐positive bacteria (*S. aureus* and *C. michiganensis*) and two yeasts (*C. albicans* and *Z. tritici*) determined by a classic overlay assay; the legend represents the zone of inhibition diameter in mm. (B) the bioactivity of the bacterial strains against 4 filamentous fungi (*F. solani*, *A. alternata*, *C. cassiicola* and *G. tritici*) and the oomycete *Gl. ultimum* determined by contact antagonism assay; the legend shows the % inhibition of mycelial growth compared to a control without bacteria. Overlay (C) and contact antagonism assay (D) of *P. phytofirmans* NPcay on PEM media.

The bioactivity of the *P. phytofirmans* PsJN containing the cepacin and caryoynencin BGCs was compared to that of the native producers *B. ambifaria* BCC0191 and *B. gladioli* BCC1697, respectively, under a range of growth conditions (Figure 4). Native producers with insertional mutations in the fatty acyl‐AMP ligase gene of both the cepacin (*B. ambifaria* BCC0191::*ccnJ*) (Mullins et al., 2019) and caryoynencin BGCs (*B. gladioli BCC1697*::*cayA*) (Jones et al., 2021), were also included in the assays as negative production controls. Trimethoprim selection was maintained in all experiments (except the *S. aureus* antagonism assays due to its sensitivity) and an empty vector control used to enable comparison of polyyne expression in wild‐type strains under this selection.

As the expected baseline, *P. phytofirmans* PsJN containing only the empty vector had no bioactivity against susceptible bacteria and fungi (Figure 4A, B). The cepacin BGC‐containing *P. phytofirmans* PsJN*_*NPcep exhibited a bioactivity spectrum that was broadly comparable to the native producer *B. ambifaria* BCC0191, against *Staphylococcus aureus*, *Zymoseptoria tritici* and *Corynespora cassiicola* when grown at BSM‐G pH 5 media both with and without 0.2% arabinose (Figure 4). This was in line with the observation that low heterologous production levels of cepacin for the BGC under the control of the native promoter occurred in the heterologous hosts (Figure 3B). However, *P. phytofirmans* PsJN containing the cepacin BGC under control of the Pbad promoter exhibited comparable bioactivity (Figure 4) to the native cepacin producer *B. ambifaria* BCC0191 EV, demonstrating that greater heterologous expression in *Paraburkholderia* can be achieved if the BGC is placed under the control of a suitable promoter.

The native cepacin producer *B. ambifaria* BCC0191, possessed broad antibacterial and antifungal activity under all conditions, reflecting antagonism as a result of the production of the polyyne and other antimicrobials (Figure 4). This was demonstrated by the insertional mutant, BCC0191*::ccnJ* still possessing residual bioactivity (Figure 4), corroborating previous results that the strain possesses multiple BGCs encoding potential antimicrobial metabolites (Mullins et al., 2019). The bioactivity gained by *P. phytofirmans* PsJN containing the caryoynencin BGC was much broader than that seen with the strain containing the cepacin BGC, matching the overall bioactivity spectrum of the native producer *B. gladioli* BCC1697 EV, but not showing the same outright antagonism levels against the bacterial and fungal susceptibility test organisms (Figure 4). Disruption of the caryoynencin BGC in the native *B. gladioli* host (*B. gladioli* BCC1697*::cayA*) did not eliminate bioactivity, correlating to previous analysis demonstrating the strain produces multiple antimicrobials (Jones et al., 2021). Overall, the successful introduction of potent antagonism towards a range of bacterial and fungal plant pathogens by heterologous expression of polyyne BGCs in *P. phytofirmans* PsJN validated that our strategy to create novel *Paraburkholderia* biocontrol strains is feasible.

### Temperature‐dependent differential growth of *Paraburkholderia* strains

Following the successful heterologous expression of polyyne BGCs in *P. phytofirmans* PsJN as model plant beneficial *Paraburkholderia* strain (Sessitsch et al., 2005), we screened a panel of 42 additional *Paraburkholderia* (representing 27 further species; Table S2) for preferential growth at 30°C over 37°C (Figure S7). This was carried out to characterise additional *Paraburkholderia* for use as heterologous expression hosts and specifically identify those with reduced potential for opportunistic pathogenicity.

**FIGURE 5:**
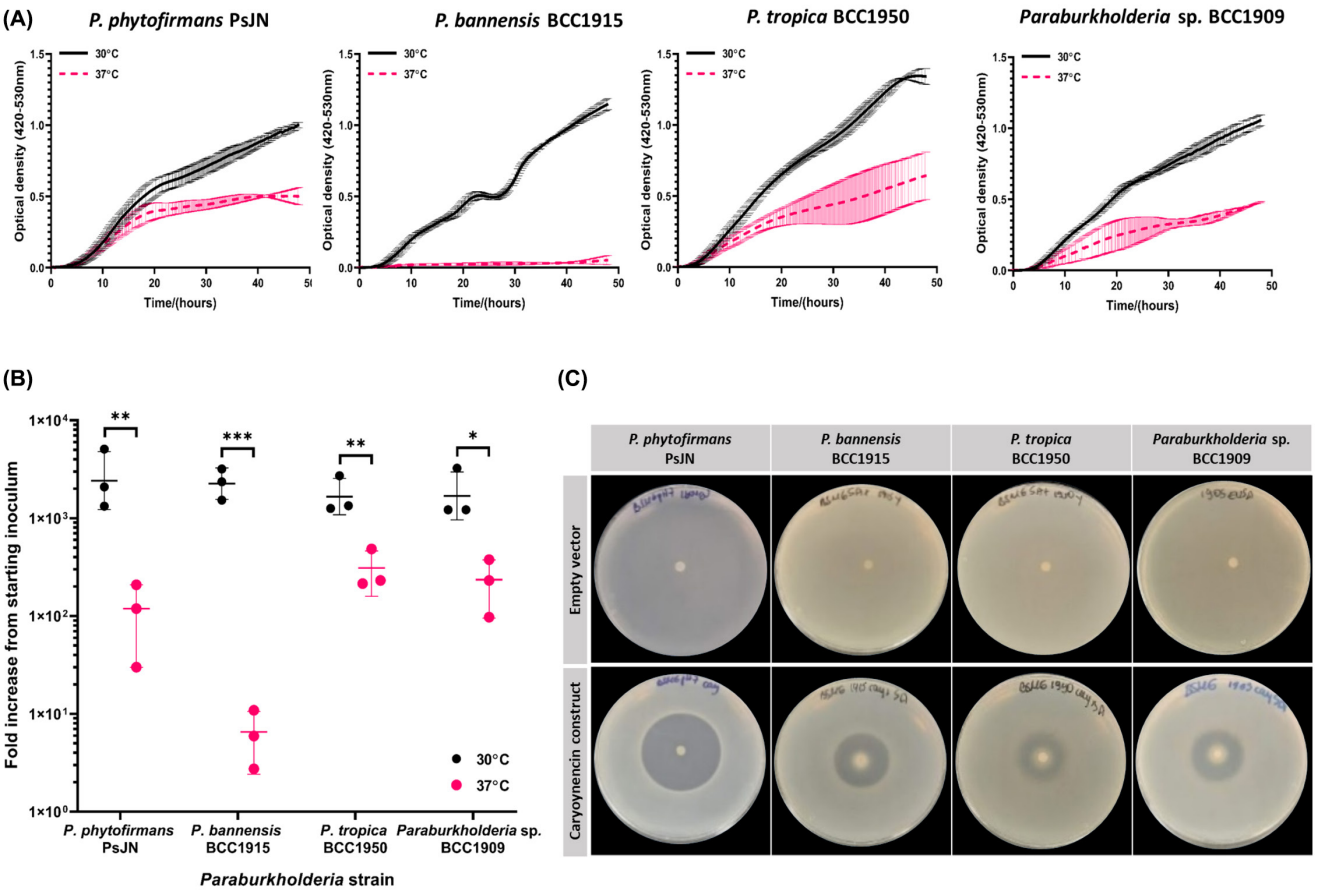
Temperature differential growth of *Paraburkholderia* strains and bioactivity. (A) the growth curves of four *Paraburkholderia* strains identified by screening to grow preferentially at 30°C over 37°C. growth was performed in BSM‐G within a bioscreen C automated growth analysis instrument (see supplementary information). (B) Viable count fold increase from starting inoculum for the four *Paraburkholderia* strains exhibiting temperature differential growth. The fold increase from starting inoculum as determined by viable count is higher at 30°C than 37°C for all four bacteria. Statistical significance (***), (**) and (*) corresponding *p* < 0.001, *p* < 0.01 and *p* < 0.05 was determined by unpaired t‐test in GraphPad prism 9.0.2. The horizontal lines represent the mean, the data points the result of each independent experiment (*n* = 3) and error bars the standard deviation. (C) the bioactivity of the four *Paraburkholderia* strains (grown at 22°C) containing the caryoynencin construct against *S. aureus* on BSM‐G media.

Out of the total of 43 *Paraburkholderia* (including *P. phytofirmans*) strains screened, 13 had significantly (*p* < 0.01) lower growth at 37°C compared to 30°C (Figure S6). The carrying capacity (K) and area under the growth curve (AUC) were evaluated using a two‐way ANOVA with Fisher LSD post hoc test to determine growth differences (see Supplementary Information). Reduced capacity for growth at 37°C was observed in 9 of the 27 *Paraburkholderia* species examined and was a common phenotype for 2 of the 3 *P. bannensis* strains evaluated (Figure 5A and Figure S6). Seven of the strains with defective growth at 37°C were characterised reference strains (*P. aspalathi* LMG 27731^T^, *P. caffeinilytica* LMG 28690^T^, *P. gisengisoli* LMG 24044^T^, *P. piptadeniae* LMG 29163^T^, *P. caledonica* LMG 19076^T^, *P. caribensis* LMG 18531^T^ see Figure S8; and *P. phytofirmans* PsJN, see Figure 5A), whilst the remaining five were isolated from the Bornean jungle, Sabah, Malaysia (*P. bannensis* BCC1915, *P. bannensis* BCC1914, *Paraburkholderia* sp. BCC1909, *P. tropica* BCC1950, *P. tropica* BCC1933, *P. tropica* BCC1943). The growth curves for 11 of the 13 strains showed considerable growth impairment at 37°C (Figure 5A; Figure S7), whilst a complete inhibition of growth at 37°C was seen with *P. bannensis* strain BCC1915 (Figure 5A).

Three of the *Paraburkholderia* strains with temperature preferential growth at 30°C over 37°C were selected as potential heterologous hosts for polyyne BGC expression: *P. bannensis* BCC1915, *P. tropica* BCC1950 and *Paraburkholderia* sp. BCC1909 (Figure 5A). All three were genetically amenable to mobilisation of the empty vector and the construct containing the caryoynencin BGC, including *P. bannensis* BCC1915. This strain had no observable growth at 37°C, but possessed a normal sigmoidal growth at 30°C (Figure 5A). Examination of cellular viability measured at the 48‐h end‐point of the growth curve, demonstrated that it was significantly higher for all three heterologous hosts strains at 30°C than at 37°C, with a 3‐log difference seen for *P. bannensis* BCC1915 (Figure 5B). Caryoynencin was produced by all three novel *Paraburkholderia* heterologous hosts (see LC–MS data within the Supplementary Information; Figure S4F, G, H) and they were bioactive against *S. aureus* (Figure 5C) and the oomycete *Gl. ultimum* (Figure S8).

## DISCUSSION

Heterologous expression of specialised metabolite BGCs from *Burkholderia* in a suitable heterologous host is the first step towards engineering a biocontrol strain for use as a safe biopesticide. This study achieved cloning and heterologous expression of the cepacin and caryoynencin BGCs in *B. ambifaria* and *P. phytofirmans*. In addition, by using the arabinose‐inducible Pbad promoter as a proof of principle, it was shown that a non‐native promoter can drive the expression of the biosynthetic genes in the polyyne BGCs. The caryoynencin BGC was also expressed in three novel environmental *Paraburkholderia* strains with considerably reduced ability to grow at 37°C, providing an avenue for engineering of safer, non‐pathogenic strains for biotechnological applications.

### Pbad promoter regulation by arabinose assessed by *luxCDABE* reporter

pMLBAD was chosen as a vector for this study following successful use in cloning and gene expression in *E. coli* and *B. cepacia* complex bacteria (Lefebre & Valvano, 2002). It contains the broad host pBBR1 origin of replication from *Bordetella bronchiseptica* (Antoine & Locht, 1992) and a mobilisation domain, mob, encoding for a relaxase, enabling conjugal transfer (Szpirer et al., 2001). The pBBR1 replicon is maintained at around five copies per cell in *E. coli*, making pMLBAD a low‐copy plasmid allowing for expression similar to chromosomal levels (Jahn et al., 2016) for this host bacterium. The copy number of pMLBAD in *Burkholderia* and *Paraburkholderia* species was previously unknown. Our study determined the copy number of the pMLBAD plasmid to be variable (between 2–19 copies/cell) for the *Burkholderiales*, depending on the cloned construct, strain and host species background. Overexpression of gene clusters due to high plasmid copy number is a limitation of this approach to heterologous expression. However, given that both polyyne clusters successfully expressed cepacin and caryoynencin using the pMLBAD vector in *Paraburkhoderia* and *B. ambifaria* across a range of 3 to 11 in the copy number, respectively, it suggests a range of tolerance for this plasmid system and the cloned polyyne pathways. The Pbad promoter is rapidly activated by the addition of 0.2% (w/v) arabinose and repressed by 0.2% (w/v) glucose to achieve tight transgene regulation as determined by using an alkaline phosphatase reporter in *E. coli* (Guzman et al., 1995). The use of pMLBAD‐eGFP in *B. cepacia* complex species showed activation of Pbad by arabinose from the baseline by the addition of L‐arabinose at concentrations 0.2% and 2.0% (w/v), as assessed by a fluorescent assay (Lefebre & Valvano, 2002).

However, the supposed repression of the promoter by glucose was not as apparent in the assay and the study concluded that the observation was due to changes in cell metabolism due to the different carbon sources and potential auto‐fluorescence (Lefebre & Valvano, 2002). We show that whilst the Pbad promoter is activated by arabinose and suppressed by glucose in *E. coli*, the suppression by glucose was not achieved in the *Burkholderiales* strains evaluated. *Burkholderiales* are known to have extensive, paralogous pathways for the catabolism of different carbon sources (Chain et al., 2006) and our data support that repression of these pathways by glucose as observed in *E. coli*, may not always hold true. Furthermore, our findings demonstrate that the addition of higher arabinose concentrations did not lead to greater promoter activation, as measured by the luminescence intensity, supporting the previous reports of all‐or‐none regulation of Pbad (Khlebnikov et al., 2000). The difference in induction responses between *E. coli* and the *Burkholderia* species examined in this study may be down to difference in the consumption of arabinose and the regulatory responses this metabolism subsequently elicits. Different responses to arabinose presence were seen in *B. ambifaria* where cepacin production increased (Figure 3B), compared to *B. gladioli* where caryoynencin production was repressed, which supports the fact that regulation of BGCs in *Burkholderia* is complex and variable.

### Polyyne BGC cloning using in vivo yeast recombination

Our study cloned and expressed two *Burkholderia* polyyne BGCs in yeast‐adapted shuttle vectors using a homologous recombination cloning approach. The in vivo homology recombination assembly in *S. cerevisiae* has been used to clone and express heterologous bacterial pathways in model organisms like *Streptomyces* (Bauman et al., 2019) and *E. coli* (Schimming et al., 2014). Although BGCs from *Burkholderia* have been cloned by RecE/RecT mediated linear–linear homologous recombination in (LLHR) and heterologously expressed in *E. coli* (Thongkongkaew et al., 2018), there are currently no reports of cloning *Burkholderia* BGCs using in vivo homology recombination in yeast. Unlike *E. coli* and *Streptomyces*, *Burkholderia* and *Paraburkholderia* species have not been extensively used and characterised as heterologous hosts. A recent study reported the heterologous expression of a lasso peptide capstruin in a *Burkholderia* heterologous host with production 60–500× higher than in *E. coli* host (Kunakom & Eustaquio, 2020). This is not surprising given the differences in GC content, codon usage, regulatory elements and level of post‐translational modification that influence the ability of the heterologous host to produce the BGC of interest (Zhang et al., 2019). The low stability of the pMLBAD‐based polyyne encoding constructs in the absence of antibiotic selection (Figure S5) would likely prove insufficient for agricultural applications. Future work in relation to biopesticidal applications would need to consider stable cloning via integrative vector constructs from which other markers such as antibiotic resistance cassettes could be removed.

### Polyyne BGC heterologous expression and in vitro bioactivity evaluation

The level of production of caryoynencin by all three heterologous hosts (*B. ambifaria* BCC1105, *B. ambifaria* BCC0191 and *P. phytofirmans* PsJN) was comparable to the native *B. gladioli* BCC1697 producer on our biomimetic pea exudate medium. Additionally, the caryoynencin‐producing *P. phytofirmans* PsJN host had a similar in vitro bioactivity against a range of fungal, oomycete and bacterial plant pathogens to the native *B. gladioli* producer. On the other hand, for the BGC regulated by the native promoter, cepacin was produced in lower quantities by *P. phytofirmans* PsJN and *B. ambifria* BCC1105 compared to the *B. ambifaria* BCC0191 native producer (Figure 3). This was reflected by the limited in vitro bioactivity of the *P. phytofirmans* PsJN containing the cepacin BGC against susceptible organisms (Table S3). *P. phytofirmans* PsJN expressing the cepacin BGC under the control of the arabinose‐inducible Pbad promoter produced similar levels of cepacin and comparable bioactivity to the native producer. This suggests that the heterologous hosts have the biosynthetic capability to produce cepacin, but the native promoter may not be as active in these strains. This variation was not surprising because the cepacin BGC has a quorum sensing‐dependent LuxRI‐based regulator associated with it (Mullins et al., 2019), which is absent from the caryoynencin BGC. Overall, the regulation and expression of polyyne BGCs within different *Burkholderia* host strains is highly variable and requires further study to evaluate key factors that alter production of these metabolites.

To our knowledge, there have been no reports of a wild type *Burkholderia* strain encoding and making multiple polyynes. The successful expression of the caryoynencin BGC in *B. ambifaria* BCC0191, a native cepacin producer, was a unique achievement of this study. It is interesting to note that, despite the high degree of similarity between some of the enzymes encoded by the two gene clusters, no structural variants of caryoynencin and cepacin appear to be produced. This suggests that the two pathways function completely independently, and no crossover of homologous enzymes between pathways occurs to drive the formation of novel polyynes or shunt metabolites. This biosynthetic independence opens up the possibility of building heterologous hosts containing multiple polyyne and other specialised metabolite BGCs.

### *Paraburkholderia* as heterologous expression hosts and less pathogenic biocontrol strains

A key concern about the use of *Burkholderia* as biopesticides is the opportunistic pathogenicity of multiple species and the potential health threat to vulnerable individuals (Parke & Gurian‐Sherman, 2001). *Paraburkholderia*, a newly classified genus of predominantly non‐pathogenic, environmental *Burkholderiales* (Sawana et al., 2014) could offer the opportunity for engineering a ‘safer’ biopesticide based on heterologous production of antimicrobial *Burkholderia* metabolites. *P. phytofirmans* PsJN used in this study does not produce known antimicrobial metabolites, and LC–MS analyses indicate that very few specialised metabolites are produced by this strain, suggesting it offers strong potential to be developed into a broadly applicable heterologous host. *P. phytofirmans* PsJN stimulates the production of antifungal phenolic compounds in grapevine (Miotto‐Vilanova et al., 2019) and induces resistance in *Arabidopsis thaliana* against *Pseudomonas syringae pv. tomato* by activating salicylic acid‐, jasmonate‐, and ethylene‐signalling pathways (Timmermann et al., 2017). The strain has also been reported to have plant growth‐promoting properties in tomato (Pillay & Nowak, 1997), potato (Frommel et al., 1991; Kurepin et al., 2015), grapevine (Barka et al., 2000) and *A. thaliana* (Poupin et al., 2013). These reports of the beneficial interaction of *P. phytofirmans* with plants make this bacterium an appealing species to explore for optimising heterologous expression and engineering for biocontrol purposes. Furthermore, by screening 42 additional *Paraburkholderia* for temperature‐dependent differential growth, we identified 12 strains with poor growth at 37°C compared with 30°C. Three of these novel *Paraburkholderia* successfully expressed caryoynencin and demonstrated in vitro bioactivity.

Given that either reclassification of the genus *Paraburkholderia* or the restricted growth at 37°C are not proof that strains from these taxa will not show pathogenicity towards humans, animals or plants, further work on their potential for infection is needed. Cases of *Paraburkholderia fungorum* isolation from human and veterinary sources, including human cerebrospinal fluid, vaginal secretions, sputum of CF patients, murine noses, porcine brain, and deer brain stem, have been reported (Coenye et al., 2001; Vandamme & Peeters, 2014). A case of *P. tropica* infection post‐surgery in a very young and heavily immunocompromised child was also documented and noted to have been successfully cleared through antibiotic therapy (Deris et al., 2010). However, a search of the current literature post the 2014 proposal of *Paraburkholderia* (Sawana et al., 2014) only further linked *P. fungorum* to a human skin granuloma (Zhang et al., 2014), indicating very limited cases of infection attributable to the genus are being reported in comparison to *Burkholderia* species. Testing using mammalian, non‐mammalian and plant infection models are needed to understand the exact pathogenic potential of the *Paraburkholderia* strains characterised in this study is required. In addition, the cytotoxicity of polyynes will also need to be evaluated to show these natural products do not have acute safety issues. Overall, the successful expression of polyyne pathways in *Paraburkholderia* with restricted growth at 37°C potentially opens multiple avenues for engineering safe biopesticides which require further exploration.

## AUTHOR CONTRIBUTIONS

E.M., A.B, G.L.C. and Y.D.P. contributed to conceptualisation; Y.D.P., A.J.M, G.W. and E.M. contributed to data curation; Y.D.P and J.Z. contributed to formal analysis, investigation and visualisation; E.M., A.B. and G.L.C. contributed to funding acquisition; Y.D.P., K.W., J.Z., G.W., A.J.M. and E.M. contributed to methodology; Y.D.P., G.L.C. and E.M. contributed to project administration; E.M., A.B., A.A., G.W., A.J.M. and G.L.C. contributed to resources; Y.D.P. contributed to resources and validation; E.M., G.L.C. and A.B. contributed to supervision; Y.D.P. and E.M. contributed to writing—original draft; all authors contributed to writing**—**review and editing.

## CONFLICT OF INTEREST

The authors do not declare any conflicts of interest in relation to this research or its funding.

## Supporting information


Table S1
Table S2.
Table S3.
Table S4.
Table S5.
Table S6.
Table S7.
Table S8.
Figure S1.
Figure S2.
Figure S3.
Figure S4.
Figure S5.
Figure S6
Figure S7.
Figure S8.
