# Supplementary material for: Cloning and expression of Burkholderia polyyne biosynthetic gene clusters in Paraburkholderia hosts provides a strategy for biopesticide development
Source: Microb Biotechnol. 2022 Jul 13;15(10):2547–61. doi: 10.1111/1751-7915.14106 (PMC9518984; doi:10.1111/1751-7915.14106)
Supplement: Supplementary file 1 — Table S1 Table S2. Table S3. Table S4. Table S5. Table S6. Table S7. Table S8. Figure S1. Figure S2. Figure S3. Figure S4. Figure S5. Figure S6 Figure S7. Figure S8. [file MBT2-15-2547-s001.docx]

**Supplementary Information**

**Cloning and expression of *Burkholderia* polyyne biosynthetic gene clusters in *Paraburkholderia* hosts provides a strategy for biopesticide development**

Yoana D. Petrova^1*^, Jinlian Zhao^2^, Gordon Webster^1^, Alex J. Mullins^1^, Katherine Williams^3^, Amal S. Alswat^1^, Gregory L. Challis^2,4,5^, Andrew M. Bailey^3^ and Eshwar Mahenthiralingam^1*^

^1^School of Biosciences, Cardiff University, Cardiff, U.K.; ^2^Department of Chemistry, University of Warwick, Coventry, UK; ^3^School of Biological Sciences, University of Bristol, Bristol, UK; ^4^Warwick Integrative Synthetic Biology Centre, University of Warwick, Coventry, UK; ^5^Department of Biochemistry and Molecular Biology, Biomedicine Discovery Institute, Monash University, Clayton, Australia

Contents

[**Supplementary Methods** 2](#_Toc104237948)

[**Strains and growth conditions** 2](#_Toc104237949)

[**Triparental conjugation of plasmid constructs in *Burkholderiales*** 2](#_Toc104237950)

[**Confirmation of metabolite production by heterologous host using high-resolution mass spectrometry** 2](#_Toc104237951)

[**Plasmid copy number determination by qPCR** 3](#_Toc104237952)

[**Temperature differential growth dynamics of *Paraburkholderia* panel** 3](#_Toc104237953)

[**Supplementary Tables** 5](#_Toc104237954)

[**Table S1. Bacterial and yeast strains used in this study** 5](#_Toc104237955)

[**Table S2. Reference strains and novel *Paraburkholderia* isolates** 7](#_Toc104237956)

[**Table S3. Fungal and bacterial susceptibility organisms: assay type, incubation temperature and duration** 9](#_Toc104237957)

[**Table S4. Plasmids used in this study.** 10](#_Toc104237958)

[**Table S5. Primers used in this study.** 11](#_Toc104237959)

[**Table S6. Thermal cycling PCR for fragment amplification using Q5 polymerase**. 14](#_Toc104237960)

[**Table S7. Thermal cycling PCR for colony PCR using *Taq* polymerase.** 14](#_Toc104237961)

[**Table S8. Thermal cycling for qPCR using SyGreen Mix.** 14](#_Toc104237962)

[**Supplementary Figures** 15](#_Toc104237963)

[**Figure S1. Restriction digest gel electrophoresis of the plasmid constructs.** 15](file:///C:\Users\c1814302\OneDrive%20-%20Cardiff%20University\Insertional%20mutagenesis\Heterologous%20expression%20chapter\heterologous%20expresssion%20manuscript\Manuscript_draft_1\edited_revision2\Suppl_MICROBIO-2-22-071-RA-R1%5b25708%5d.docx#_Toc104237964)

[**Figure S2. Cloning of polyyne gene clusters cepacin and caryoynencin in yeast adapted pMLBAD.** 16](file:///C:\Users\c1814302\OneDrive%20-%20Cardiff%20University\Insertional%20mutagenesis\Heterologous%20expression%20chapter\heterologous%20expresssion%20manuscript\Manuscript_draft_1\edited_revision2\Suppl_MICROBIO-2-22-071-RA-R1%5b25708%5d.docx#_Toc104237965)

[**Figure S3. Heterologous expression of cepacin by *B. ambifaria* BCC1105 and *P. phytofirmans* PsJN** 19](file:///C:\Users\c1814302\OneDrive%20-%20Cardiff%20University\Insertional%20mutagenesis\Heterologous%20expression%20chapter\heterologous%20expresssion%20manuscript\Manuscript_draft_1\edited_revision2\Suppl_MICROBIO-2-22-071-RA-R1%5b25708%5d.docx#_Toc104237966)

[**Figure S4. Heterologous expression of caryoynencin by *B. ambifaria* BCC1105, *B. ambifaria* BCC0191 and *P. phytofirmans* PsJN** 20](file:///C:\Users\c1814302\OneDrive%20-%20Cardiff%20University\Insertional%20mutagenesis\Heterologous%20expression%20chapter\heterologous%20expresssion%20manuscript\Manuscript_draft_1\edited_revision2\Suppl_MICROBIO-2-22-071-RA-R1%5b25708%5d.docx#_Toc104237967)

[**Figure S5. pMLBAD constructs copy number and stability of pMLBAD::luxCDABE** 24](file:///C:\Users\c1814302\OneDrive%20-%20Cardiff%20University\Insertional%20mutagenesis\Heterologous%20expression%20chapter\heterologous%20expresssion%20manuscript\Manuscript_draft_1\edited_revision2\Suppl_MICROBIO-2-22-071-RA-R1%5b25708%5d.docx#_Toc104237968)

[**Figure S6 Temperature differential growth of *Paraburkholderia* panel.** 25](file:///C:\Users\c1814302\OneDrive%20-%20Cardiff%20University\Insertional%20mutagenesis\Heterologous%20expression%20chapter\heterologous%20expresssion%20manuscript\Manuscript_draft_1\edited_revision2\Suppl_MICROBIO-2-22-071-RA-R1%5b25708%5d.docx#_Toc104237969)

[**Figure S7. Temperature differential growth of selected *Paraburkholderia* strains.** 26](file:///C:\Users\c1814302\OneDrive%20-%20Cardiff%20University\Insertional%20mutagenesis\Heterologous%20expression%20chapter\heterologous%20expresssion%20manuscript\Manuscript_draft_1\edited_revision2\Suppl_MICROBIO-2-22-071-RA-R1%5b25708%5d.docx#_Toc104237970)

[**Figure S8. Bioactivity of recombinant *Paraburkholderia* strains against *Gl. ultimum*.** 27](file:///C:\Users\c1814302\OneDrive%20-%20Cardiff%20University\Insertional%20mutagenesis\Heterologous%20expression%20chapter\heterologous%20expresssion%20manuscript\Manuscript_draft_1\edited_revision2\Suppl_MICROBIO-2-22-071-RA-R1%5b25708%5d.docx#_Toc104237971)

[**References** 28](#_Toc104237972)

# **Supplementary Methods**

##

## **Strains and growth conditions**

For routine growth, the *E. coli* and *Burkholderia* strains were incubated at 37°C, whilst *S. cerevisiae* and the *Paraburkholderia* strains were revived at 30°C. Following chemical transformation of *E. coli* with the plasmid constructs, LB (Luria-Bertani) media supplemented with 50 µg/mL trimethoprim or kanamycin was used for selection. Where minimal media were required, M9 (Atlas, 2010) was used for the *E. coli* and basal salt media with glycerol (BSM-G) (Hareland *et al.*, 1975, Mahenthiralingam *et al.*, 2011) for *Burkholderia* and *Paraburkholderia.* Pea exudate media (PEM) was prepared as described (Mullins *et al.*, 2021). A collection of 19 environmental *Paraburkholderia* was assembled after sampling the rhizosphere and soil of forest floor plants in Bornean Jungle as described (Alswat, 2020). The sampling and initial culture of this collection was carried out with permission from the Sabah Wildlife Departmental, Sabah, Malaysia, and took place in August 2008, prior to the Nagoya protocol. All experiments were performed in at least three independent biological replicates unless stated otherwise.

## **Triparental conjugation of plasmid constructs in *Burkholderiales***

All the constructs were mobilised in *Burkholderia* and *Paraburkholderia* using the triparental conjugation mating with *E. coli* DH5α containing the construct of interest and *E. coli* HB101 containing the helper plasmid pRK2013 (Craig *et al.*, 1989) (Table S3). The selection conditions were 150 µg/mL trimethoprim and 600 units/mL polymyxin for the *Burkholderia* species, *P. phytofirmans* and *P. bannensis* BCC1915. The transconjugants selection for *Paraburkholderia* BCC1909 and *P. tropica* BCC1950 was performed using modified basal salt media with 0.2% (w/v) sodium citrate as the sole carbon source. In all cases successful conjugation was confirmed by colony PCR with the same primers used to confirm the presence of the constructs in *E. coli* (Table S6). The identity of the transconjugants was confirmed by Sanger sequencing (Eurofins, UK) of the 16S rRNA gene, amplified with the 27F and 1492R universal primers (Table S6).

## **Confirmation of metabolite production by heterologous host using high-resolution mass spectrometry**

All strains were grown at 30°C overnight in LB medium (with trimethoprim for the heterologous hosts) and then inoculated onto BSM-G agar plates supplemented with 0.2% L-arabinose (for Pbad constructs) and trimethoprim. After incubation at 22°C for three days, the medium in single plate was cut into small pieces after removing cells and extracted with 10 mL of ethyl acetate (EtOAc) for 2 hours, followed by evaporating under rotary vacuum and re-dissolving in 1 mL of 50% acetonitrile in water. The crude extracts were then analysed by UHPLC-ESI-Q-TOF-MS after centrifugation to remove debris. UHPLC-ESI-Q-TOF-MS analysis were performed using a Dionex UltiMate 3000 UHPLC connected to a Zorbax Eclipse Plus C-18 column (100 × 2.1 mm, 1.8 μm) coupled to a Bruker Compact mass spectrometer. Mobile phases consisted of water and acetonitrile (ACN), each supplemented with 0.1% formic acid. After 5 minutes of isocratic run at 5% ACN, a gradient of 5% to 100% ACN in 12 minutes was employed with flow rate 0.2 ml min−1, followed by keeping constant for 5 minutes and then returning to initial conditions within 3 minutes. The mass spectrometer was operated in positive-ion mode with a scan range of 50–3,000 m/z. Source conditions were: end-plate offset at −500 V, capillary at −4,500 V, nebulizer gas (N2) at 1.6 bar, dry gas (N2) at 81 min−1 and dry temperature at 180 °C. Ion transfer conditions were: ion funnel radio frequency (RF) at 200 Vpp, multiple RF at 200 Vpp, quadrupole low mass at 55 m/z, collision energy at 5.0 eV, collision RF at 600 Vpp, ion cooler RF at 50–350 Vpp, transfer time at 121 μs and pre-pulse storage time at 1 μs. Calibration was performed with 1 mM sodium formate through a loop injection of 15 μL at the start of each run.

## **Plasmid copy number determination by qPCR**

Plasmid copy number of the polyyne and empty vector constructs in the heterologous hosts was estimated by quantitative PCR performed using Agilent Mx3000P qPCR System (Table S6 and Table S9). Total DNA of each strain was extracted from an overnight culture in Tryptic Soya Broth (TSB) supplemented with 50 µg/mL trimethoprim using the Maxwell® 16 Tissue DNA Purification Kit; 2 ng DNA was used as template in the qPCR reaction. Two sets of qPCR primers were used: one set targeting a 143 bp portion of the chromosomally encoded single copy *rpoD* gene (Ogier *et al.*, 2019) and set targeting 173 bp from the 2µ region of the pMLBAD_yeast plasmid. The copy number of the *rpoD* gene and 2µ was determined from a standard curve and plasmid copy number calculated by dividing the 2µ by the *rpoD* copy number. The standard curve was generated using 1011 bp *rpoD* standard, amplified from the genomic DNA of *Burkholderia cenocepacia* J2315, and 1115 bp 2µ standard, amplified from purified pMLBAD_yeast vector; amplification efficiencies for both were 91-96%.

##

## **Temperature differential growth dynamics of *Paraburkholderia* panel**

Master plates of the *Paraburkholderia* strains panel (Table S2) was prepared by growing the strains in a 96-microwell plate format in TSB for 20 hours at 30°C on a rocking platform (50 rpm); 8% DMSO was added as a cryoprotectant and the plates frozen at -80°C until required for experiments. Prior to each growth experiment, a master plate was thawed, strains diluted 1:100 in BSM-G media and incubated at 30°C for 20 hours in a 96-microwell flat bottom plate; each master plate was subjected to maximum of 3 freeze-thaw cycles. Fresh BSM-G media was inoculated with ~ 1 x 10^6^ cfu/mL from the overnight cultures and 48-hour growth dynamics for the strain panel determined at both 30°C and 37°C using Bioscreen C instrument. Optical density readings at 420-530 nm were taken every 15 minutes, with 10 second orbital shaking prior to each reading.

# **Supplementary Tables**

## **Table S1. Bacterial and yeast strains used in this study**

| **Strain** | **Genotype or Source Description** | **Source/Reference** |
| --- | --- | --- |
| *Saccharomyces cerevisiae* YPH500 | MATalpha ura3-52 lys2-801 ade2-101 trp1-delta63 his3-delta200 leu2-delta1 | Pahirulzam *et al.,*2012 |
| *Escherichia coli* HB101 pRK2013 | F−, hsdS20(r−Bm−B), recA13, ara14, proA2, lacY1, galK2, rpsL20 (Smr) xyl-5, mtl-1, supE44, γ-, Knr | Figurski and Helinski, 1979 |
| *Escherichia coli* DH5α | F- φ80lacZ∆M15 ∆(lacZYA-argF) U169 recA1 endA1 hsdR17 (rk-, mk+) phoA supE44 λ- thi-1 gyrA96 relA1 | Invitrogen MAX Efficiency™ DH5α Competent Cells |
| *Burkholderia vietnamiensis* G4 | Isolated from wastewater in the USA | ATCC 53617 |
| *Burkholderia gladioli* BCC1697 | Isolated from a cystic fibrosis patient in USA | Jones *et al.,* 2021 |
| *Burkholderia gladioli* BCC1697::*cayA* | *Burkholderia gladioli*BCC1697 caryoynencin mutant | Jones et al., 2021 |
| *Burkholderia gladioli* BCC1697_EV | *Burkholderia gladioli* BCC1697, pMLBAD_yeast | This study |
| *Burkholderia ambifaria* BCC0191 | Environmental isolate from soil in USA; characterised biocontrol strain | Mao *et al.,* 1997 |
| *Burkholderia ambifaria* BCC0191*::ccnJ* | *Burkholderia ambifaria* BCC0191 cepacin mutant | Mullins *et al.,*2018 |
| *Burkholderia ambifaria* BCC0191 EV | *Burkholderia ambifaria* BCC0191*,*pMLBAD_yeast | This study |
| *Burkholderia ambifaria* BCC0191 NPcay | *Burkholderia ambifaria* BCC0191*,* pMLBAD_yeast_NPcay | This study |
| *Burkholderia ambifaria* BCC1105 | Environmental isolate from maize rhizosphere in Italy | Dalmastri *et al.,*1999 |
| *Burkholderia ambifaria* BCC1105 EV | *Burkholderia ambifaria* BCC1105*,*pMLBAD_yeast | This study |
| *Burkholderia ambifaria* BCC1105 Pbadcep | *Burkholderia ambifaria* BCC1105*,*pMLBAD_yeast_Pbadcep | This study |
| *Burkholderia ambifaria* BCC1105 Pbadcay | *Burkholderia ambifaria* BCC1105*,*pMLBAD_yeast_Pbadcay | This study |
| *Burkholderia ambifaria* BCC1105 NPcep | *Burkholderia ambifaria* BCC1105*,* pMLBAD_yeast_NPcep | This study |
| *Burkholderia ambifaria* BCC1105 NPcay | *Burkholderia ambifaria* BCC1105*,* pMLBAD_yeast_NPcay | This study |
| *Paraburkholderia phytofirmans* PsJN | Isolated from surface-sterilized onion roots infected with the mycorrhizal fungus *Glomus vesiculiferu* | LMG 22146^T^ |
| *Paraburkholderia phytofirmans* PsJN EV | *Paraburkholderia phytofirmans* PsJN, pMLBAD_yeast | This study |
| *Paraburkholderia phytofirmans* PsJN EV Pbadcep | *Paraburkholderia phytofirmans* PsJN, pMLBAD_yeast_Pbadcep | This study |
| *Paraburkholderia phytofirmans* PsJN EV Pbadcay | Paraburkholderia phytofirmans PsJN, pMLBAD_yeast_Pbadcay | This study |
| *Paraburkholderia phytofirmans* PsJN EV NPcep | *Paraburkholderia phytofirmans* PsJN*,* pMLBAD_yeast_NPcep | This study |
| *Paraburkholderia phytofirmans* PsJN EV Npcay | *Paraburkholderia phytofirmans* PsJN*,* pMLBAD_yeast_NPcay | This study |
| *Paraburkholderia bannensis* BCC1915 | Isolated from soil in Borneo Jungle | Alswat 2020 |
| *Paraburkholderia bannensis* BCC1915 EV | *Paraburkholderia bannensis*BCC1915, pMLBAD_yeast | This study |
| *Paraburkholderia bannensis* BCC1915 NPcay | *Paraburkholderia bannensis* BCC1915*,* pMLBAD_yeast_NPcay | This study |
| *Paraburkholderia tropica* BCC1950 | Isolated from soil in Borneo Jungle | Alswat 2020 |
| *Paraburkholderia tropica* BCC1950 EV | *Paraburkholderia tropica* BCC1950, pMLBAD_yeast | This study |
| *Paraburkholderia tropica* BCC1950 NPcay | *Paraburkholderia tropica* BCC1950, pMLBAD_yeast_NPcay | This study |
| *Paraburkholderia species* BCC1909 | Isolated from soil in Borneo Jungle | Alswat 2020 |
| *Paraburkholderia species* BCC1909 EV | *Paraburkholderia species* BCC1909, pMLBAD_yeast | This study |
| *Paraburkholderia species* BCC1909 NPcay | *Paraburkholderia species* BCC1909, pMLBAD_yeast_NPcay | This study |

## **Table S2. Reference strains and novel *Paraburkholderia* isolates**

| **Reference *Paraburkholderia* strains** | **Source Description** | **Reference** |
| --- | --- | --- |
| *Paraburkholderia aspalathi* | Isolated from root nodules of *Aspalathus abietina* Thunb legume in South Africa | LMG 27731^T^ |
| *Paraburkholderia caballeronis* | Isolated from rhizosphere of tomato in Mexico | LMG 26416^T^ |
| *Paraburkholderia caffeinilytica* | Isolated from tea plantation soil in China | LMG 28690^T^ |
| *Paraburkholderia diazotrophica* | Isolated from *Mimosa candollei* root nodule in Brazil | LMG 26031^T^ |
| *Paraburkholderia ginsengisoli* | Isolated from gingseng field in South Korea | LMG 24044^T^ |
| *Paraburkholderia kirstenboschensis* | Isolated from *Virgilia oroboides* root nodules in South Africa | LMG 28727^T^ |
| *Paraburkholderia pallidirosea* | Isolated from monsoon evergreen broad-leaved forest soil in China | LMG 28846^T^ |
| *Paraburkholderia piptadeniae* | Isolated from *Piptadenia gonoacantha* root nodule in Brazil | LMG 29163^T^ |
| *Paraburkholderia ribeironis* | Isolated from *Piptadenia gonoacantha*  root nodule in Brazil | LMG 29351^T^ |
| *Paraburkholderia dipogonis* | Isolated from *Dipogon lignosus* in New Zealand | LMG 28415^T^ |
| *Paraburkholderia caledonica* | Isolated from soil rhizosphere in Edinburgh | LMG 19076^T^ |
| *Paraburkholderia phymatum* | Isolated from root nodule of *Machaerium lunatum* in South America | LMG 21445^T^ |
| *Paraburkholderia caribensis* | Isolated from vertisol in the French West Indies | LMG 18531^T^ |
| *Paraburkholderia fungorum* | Isolated from fungus *Phanerochaete chrysosporium* in France | LMG 16225^T^ |
| *Paraburkholderia graminis* | Isolated from maize senescent root system in France. | LMG 18924^T^ |
| *Paraburkholderia mimosarum* | Isolated from *Mimosa pigra* root nodule in China. | LMG 23256^T^ |
| *Paraburkholderia phenoliruptrix* | Isolated from chemostat with 2,4,5-trichlorophenoxyacetic acid in Canada | LMG 22037^T^ |
| *Paraburkholderia sacchari* | Isolated from soil of sugar cane plantation in Brazil | LMG 19450^T^ |
| *Paraburkholderia terricola* | Isolated from agricultural soil in Belgium | LMG 20594^T^ |
| *Paraburkholderia tropica* | Isolated from sugar cane roots in Brazil | LMG 22274^T^ |
| *Paraburkholderia tuberum* | Isolated from *Aspalathus carnosa* root nodule in South Africa | LMG 21444^T^ |
| **Novel *Paraburkholderia* isolates** | **Source Description** | **Reference** |
| *Paraburkholderia* sp. BCC1909 | Bornean jungle soil isolate, Sabah, Malaysia | Alswat 2020 |
| *Paraburkholderia tropica* BCC1910 | Bornean jungle soil isolate, Sabah, Malaysia | Alswat 2020 |
| *Paraburkholderia tropica* BCC1911 | Bornean jungle soil isolate, Sabah, Malaysia | Alswat 2020 |
| *Paraburkholderia* sp. BCC1913 | Bornean jungle soil isolate, Sabah, Malaysia | Alswat 2020 |
| *Paraburkholderia bannensis* BCC1914 | Bornean jungle soil isolate, Sabah, Malaysia | Alswat 2020 |
| *Paraburkholderia bannensis* BCC1915 | Bornean jungle soil isolate, Sabah, Malaysia | Alswat 2020 |
| *Paraburkholderia bannensis* BCC1916 | Bornean jungle soil isolate, Sabah, Malaysia | Alswat 2020 |
| *Paraburkholderia* sp. BCC1918 | Bornean jungle soil isolate, Sabah, Malaysia | Alswat 2020 |
| *Paraburkholderia tropica* BCC1924 | Bornean jungle soil isolate, Sabah, Malaysia | Alswat 2020 |
| *Paraburkholderia tropica* BCC1925 | Bornean jungle soil isolate, Sabah, Malaysia | Alswat 2020 |
| *Paraburkholderia tropica* BCC1926 | Bornean jungle soil isolate, Sabah, Malaysia | Alswat 2020 |
| *Paraburkholderia tropica* BCC1927 | Bornean jungle soil isolate, Sabah, Malaysia | Alswat 2020 |
| *Paraburkholderia tropica* BCC1928 | Bornean jungle soil isolate, Sabah, Malaysia | Alswat 2020 |
| *Paraburkholderia tropica* BCC1929 | Bornean jungle soil isolate, Sabah, Malaysia | Alswat 2020 |
| *Paraburkholderia tropica* BCC1933 | Bornean jungle soil isolate, Sabah, Malaysia | Alswat 2020 |
| *Paraburkholderia* sp. BCC1940 | Bornean jungle soil isolate, Sabah, Malaysia | Alswat 2020 |
| *Paraburkholderia tropica* BCC1943 | Bornean jungle soil isolate, Sabah, Malaysia | Alswat 2020 |
| *Paraburkholderia tropica* BCC1945 | Bornean jungle soil isolate, Sabah, Malaysia | Alswat 2020 |
| *Paraburkholderia tropica* BCC1950 | Bornean jungle soil isolate, Sabah, Malaysia | Alswat 2020 |
| *Paraburkholderia* sp. BCC1953 | Bornean jungle soil isolate, Sabah, Malaysia | Alswat 2020 |
| *Paraburkholderia* sp. BCC1954 | Bornean jungle soil isolate, Sabah, Malaysia | Alswat 2020 |

**Table S3. Fungal and bacterial susceptibility organisms: assay type, incubation temperature and duration**.

| **Species name** | **Alternative name** | **Source/ID Number** | **Assay type** | **Incubation Temperature (°C)** | **Length of incubation (days)** |
| --- | --- | --- | --- | --- | --- |
| *Staphylococcus aureus* | - | NCTC 12981 | Overlay assay | 37 | 1 |
| *Candida albicans* | *-* | SC 5314 | Overlay assay | 37 | 1 |
| *Clavibacter michiganensis* | *-* | DSM 46364 | Overlay assay | 30 | 2 |
| *Zymoseptoria tritici* L951 | *Septoria tritici, Mycosphaerella graminicola* | Tom Hunter, Long Ashton Research Station | Overlay assay | 22 | 3 |
| *Globisporangium ultimum (Trow) Uzuhashi, Tojo & Kakishima var. ultimum* | *Pythium ultimum* | MUCL 16164 | Contact antagonism assay | 22 | 3 |
| *Fusarium redolens Wollenweber* | *Fusarium solani* | MUCL 14247 | Contact antagonism assay | 22 | 6 |
| *Alternaria alternata (Fries : Fries) von Keissler* | *Alternaria tenuis Nees* | MUCL 36 | Contact antagonism assay | 22 | 6 |
| *Corynespora cassiicola* | - | Provided by Dr Andy Bailey, School of Biological Sciences, University of Bristol, UK | Contact antagonism assay | 22 | 6 |
| *Gaeumannomyces tritici* Gt19LH(4)19d2 | *Gaeumannomyces graminis var. tritici* | Provided by Department of Biointeractions and Crop Protection, Rothamsted Research, UK | Contact antagonism assay | 22 | 6 |

## **Table S4. Plasmids used in this study.**

| **Plasmids** | **Description** | **Source/Reference** |
| --- | --- | --- |
| pMLBAD | pBBR1 ori, *araC-PBAD,*Tp^r^ , *mob^+^* | Valvano 2002 |
| pE-YA | pUC ori, 2 micron ori, *URA3*, Kan^r^ | Pahirulzam *et a.,* 2012 |
| mini-Tn5 luxCDABE | pUTmini-Tn5, *luxCDABE,* Tet^r^ | Winson *et al*., 1998 |
| pKD4 | ori_6RK_, FRT-Kan-FRT, Amp^r^ | Datsenko *et al*., 2000 |
| pMLBAD_yeast | pMLBAD, 2 micron, *URA3* | This study |
| pMLBAD_yeast_luxCDABE | pMLBAD, 2 micron, *URA3*, *luxCDABE* | This study |
| pMLBAD_yeast_luxCDABE_rev | pMLBAD, 2 micron, *URA3*, *luxCDABE*(rev) | This study |
| pMLBAD_yeast_Pbadcep | pMLBAD, 2 micron, *URA3*, cepacin BGC | This study |
| pMLBAD_yeast_Pbadcay | pMLBAD, 2 micron, *URA3*, caryoynencin BGC | This study |
| pMLBAD_yeast_NPcep | pMLBAD∆*araC-Pbad,*2 micron, *URA3*,FRT-Kan-FRT,cepacin BGC | This study |
| pMLBAD_yeast_NPcay | pMLBAD∆*araC-Pbad,*2 micron, *URA3*,FRT-Kan-FRT, caryoynencin BGC | This study |

## **Table S5. Primers used in this study.**

| **Primers** | **Reference** | **Product size (bp)** | **Primer use** | **Annealing temperature (Polymerase)** |
| --- | --- | --- | --- | --- |
| Yeast_fwd 5'-TCCGACTCGTCCAACATCAATACAACCTATCCTGGCAGTTCCCTACTCTC 3' Yeast_rev 5'- TTGATAACCTTATTTTTGACGAGGGGAAATCCAACTGATCTTCAGCATCTTTTAC-3' | This study | 2867 | To generate 2µ and URA3 yeast fragment fragment. | 66°C (Q5) |
| luxCD_fwd 5'- CCATACCCGTTTTTTTGGGCTAGCAGGAGGGCTTGGAGGATACGTATGAC-3'  luxCD_rev 5'   - GTCCTTATATTGCTATTTGAGTGATAGAATATCTCAATAGATTTTAAGACAG-3' | This study | 2415 | To generate luxCD fragment. | 61°C (Q5) |
| luxABE_fwd 5'- TGAGATATTCTATCACTCAAATAGCAATATAAGGACTCTC-3' luxABE_rev 5'- AAAATCTTCTCTCATCCGCCAAAACAGCCATCAACTATCAAACGCTTCGGTT-3' | This study | 3397 | To generate luxABE fragment. | 61°C (Q5) |
| luxABE_REV_fwd 5'-CCATACCCGTTTTTTTGGGCTAGCAGGAGGTCAACTATCAAACGCTTCGG-3' luxABE_REV_rev 5'- TGAGATATTCTATCACTCAAATAGCAATATAAGGACTC-3' | This study | 3398 | To generate luxABE(rev) fragment. | 58.5°C (Q5) |
| luxCD_REV_fwd 5'- ATATTGCTATTTGAGTGATAGAATATCTCAATAGATTTTAAGAC-3' luxCD_REV_rev 5'- GTCCTTATATTGCTATTTGAGTGATAGAATATCTCAATAGATTTTAAGACAG-3' | This study | 2466 | To generate luxCD(rev) fragment. | 58.5°C (Q5) |
| F1_cep_fwd 5'-CCATACCCGTTTTTTTGGGCTAGCAGGAGGGAAGGTGAAATGACGCTTCGAGTG-3' F1_cep_rev 5'-CTCTCTTTGCTACAGGAGTGGCGTTGTGGCACACC-3' | This study | 4100 | To generate fragment 1 cepacin. | 66°C (Q5) |
| F2_cep_fwd 5'-GCCACAACGCCACTCCTGTAGCAAAGAGAGGAATCATGTTTATC-3' F2_cep_rev 5'-AAGACAGCGTCGATGAAAATCGGGGATGCGGTTCG-3' | This study | 5281 | To generate fragment 2 cepacin. | 66°C (Q5) |
| F3_cep_fwd 5'-CGCATCCCCGATTTTCATCGACGCTGTCTTTCTTC-3' F3_cep_rev 5'-AAAATCTTCTCTCATCCGCCAAAACAGCCATCCGTTTGGGTCAAATCGATATC-3' | This study | 4602 | To generate fragment 3 cepacin. | 66°C (Q5) |
| F1_cay_fwd 5'-CCATACCCGTTTTTTTGGGCTAGCAGGAGGGCCGACATGACACATCTCGAAGTC-3' F1_cay_rev 5'-GCCATCGGTTCGCTCCTTGGTGGGCCTCAGGCCTT-3' | This study | 4182 | To generate fragment 1 caryoynencin. | 66°C (Q5) |
| F2_cay_fwd 5'-CTGAGGCCCACCAAGGAGCGAACCGATGGCAACCATC-3' F2_cay_rev 5'-TGGCTTGCTTGGCCGTCCTCGCCGGGATCATGCGT-3' | This study | 4691 | To generate fragment 2 caryoynencin. | 66°C (Q5) |
| F3_cay_fwd 5'-TGATCCCGGCGAGGACGGCCAAGCAAGCCACGCGA-3' F3_cay_rev 5'-AAAATCTTCTCTCATCCGCCAAAACAGCCATCATGGGGCCGGGGGATCCT-3' | This study | 4316 | To generate fragment 3 caryoynencin. | 66°C (Q5) |
| Kan_cep_fwd 5'-CAAGCCGTCAATTGTCTGATTCGTTACCAATGTGTAGGCTGGAGCTGCTTC-3' Kan_cep_rev 5'-GGGCTTTTATTCTGAATGGGAATTAGCCATGGTCCATATG-3' | This study | 1541 | To generate kanamycin fragment for cepacin promoter swap | 67°C (Q5) |
| NPcep_fwd 5'-ATGGCTAATTCCCATTCAGAATAAAAGCCCCAGCATCGC-3' NPcep_rev 5'-GATACCGGTTACTACCACTCGAAGCGTCATTTCACCTTCCCCCTTCGGGC-3' | This study | 2723 | To generate new promoter cepacin fragment | 67°C (Q5) |
| Kan_cay_fwd 5'-CAAGCCGTCAATTGTCTGATTCGTTACCAATGTGTAGGCTGGAGCTGCTTC-3' Kan_cay_fwd 5'-AGGCCGCCCTACCCGATGGGAATTAGCCATGGTCCATATG-3' | This study | 1542 | To generate kanamycin fragment for caryoynencin promoter swap | 67°C (Q5) |
| NPcay_fwd 5'-ATGGCTAATTCCCATCGGGTAGGGCGGCCTGCGCA-3' NPcay_rev 5'-CGCCGGGCTTGCGACTTCGAGATGTGTCATCACGGGAGGCTCCTTGGGGAACAC-3' | This study | 1059 | To generate new promoter caryoynencin fragment | 68°C (Q5) |
| Yeast_conf_fwd 5'-TATCCTGGCAGTTCCCTACTCTC- 3'  Yeast_conf_rev 5'- CCAACTGATCTTCAGCATCTTTTAC-3' | This study | 2810 | To confirm pMLBAD_yeast | 56°C (*Taq*) |
| Pbad_fwd 5'-ATGCCATAGCATTTTTATCC-3' Lux_conf_rev 5'-TGATAGAATATCTCAATAGATTTTAAGACAG-3' | This study | 2520 | To confirm pMLBAD_yeast_luxCDABE | 58°C (*Taq*) |
| Pbad_fwd 5'-ATGCCATAGCATTTTTATCC-3' Lux_REV_rev 5'-GGAGTATGTTGATAAGTTGA-3' | This study | 2296 | To confirm pMLBAD_yeast_luxCDABE_rev | 50°C (*Taq*) |
| Pbad_fwd 5'-ATGCCATAGCATTTTTATCC-3' Cep_rev 5'-TTCCGTACCCGATGATTTCC-3' | This study | 927 | To confirm pMLBAD_yeast_Pbadcep | 58°C *(Taq*) |
| Pbad_fwd 5'-ATGCCATAGCATTTTTATCC-3' Cay_rev 5'-CGATATCCTGAGGATAGAGATT-3' | This study | 1617 | To confirm pMLBAD_yeast_Pbadcay | 50°C (*Taq*) |
| NPcep_conf_fwd 5'-TGCATAGGGTTATCTTGCATGA-3' NPcep_conf_rev 5'-GATGAAGCGATCCATCTTCCTC-3' | This study | 1386 | To confirm pMLBAD_yeast_NPcep | 56°C (*Taq*) |
| NPcay_conf_fwd 5'-TGGATTCATCGACTGTGGCCGG-3' NPcay_conf_rev 5'-CGCGAATCGGTCAGGATCAGCT-3' | This study | 1911 | To confirm pMLBAD_yeast_NPcay | 64°C (*Taq*) |
| rpoD_std_fwd 5'-GATCTTGCACATCGTCGTC-3'  rpoD_std_rev 5'- GTTCGTAACGGAGACGCTG -3' | Sass *et al*., 2013 | 1011 | To prepare rpoD qPCR standard. | 59°C (*Taq)* |
| 2µ_std_fwd 5'-GGAGAAAACTCACCGAGGCA -3'  2µ_std_rev 5'-CCCGGTTCATTTTCTGCGTT -3' | This study | 1115 | To prepare 2µ qPCR standard. | 60°C (*Taq)* |
| rpoD_qPCR_fwd 5'-GAGATGAGCACCGATCACAC-3'  rpoD_qPCR_rev 5'- CCTTCGAGGAACGACTTCAG -3' | Sass *et al*., 2013 | 143 | To determine rpoD copy number by qPCR | 65°C (*SyGreen*) |
| 2µ_qPCR_fwd 5'-TGCGCAATCCACATCGGTAT -3'  2µ_qPCR_rev 5'-TGCACTGTAGGTCCGTTAAGGTT -3' | This study | 173 | To determine 2µ copy number by qPCR | 65°C (*SyGreen*) |
| 27F 5' AGAGTTTGATCCTGGCTCAG-3'  1492R 5’-GGTTACCTTGTTACGACTT-3’ | Webster *et al.*, 2004 | variable | Amplification of the 16S RNA gene | 51°C (Taq) |

## **Table S6. Thermal cycling PCR for fragment amplification using Q5 polymerase**.

| **PCR step** | **Temperature (°C)** | **Time (s)** | **Number of cycles** |
| --- | --- | --- | --- |
| Initial denaturation | 98 | 30 | 1 |
| Denaturation | 98 | 10 |  |
| Primer annealing | Variable^a^ | 30 | 40 |
| Extension | 72 | 90-180^b^ |  |
| Final extension | 72 | 120 | 1 |

^a^ Table S2 contains specific annealing temperatures

^b^ 90s for products up to 3kb, 120s for products 3-4kb, 180s for products > 4kb

## **Table S7. Thermal cycling PCR for colony PCR using *Taq* polymerase.**

| **PCR step** | **Temperature (°C)** | **Time (s)** | **Number of cycles** |
| --- | --- | --- | --- |
| Initial denaturation | 95 | 300 | 1 |
| Denaturation | 95 | 30 |  |
| Primer annealing | Variable^a^ | 30 | 35 |
| Primer Extension | 72 | 60-120^b^ |  |
| Final extension | 72 | 600 | 1 |

^b^ 60s for products up to 1kb, 90s for products 1-1.5kb, 120s for products > 1.5kb

^a^ Table S2 contains specific annealing temperatures

| **qPCR step** | **Temperature (°C)** | **Time (s)** | **Number of cycles** |
| --- | --- | --- | --- |
| Initial denaturation | 95 | 120 | 1 |
| Denaturation | 95 | 15 | 40 |
| Primer annealing | 67 | 30 |  |
| Dissociation curve | 55 | 30 | 1 |
|  | 95 | 60 |  |

## **Table S8. Thermal cycling for qPCR using SyGreen Mix.**

# **Supplementary Figures**


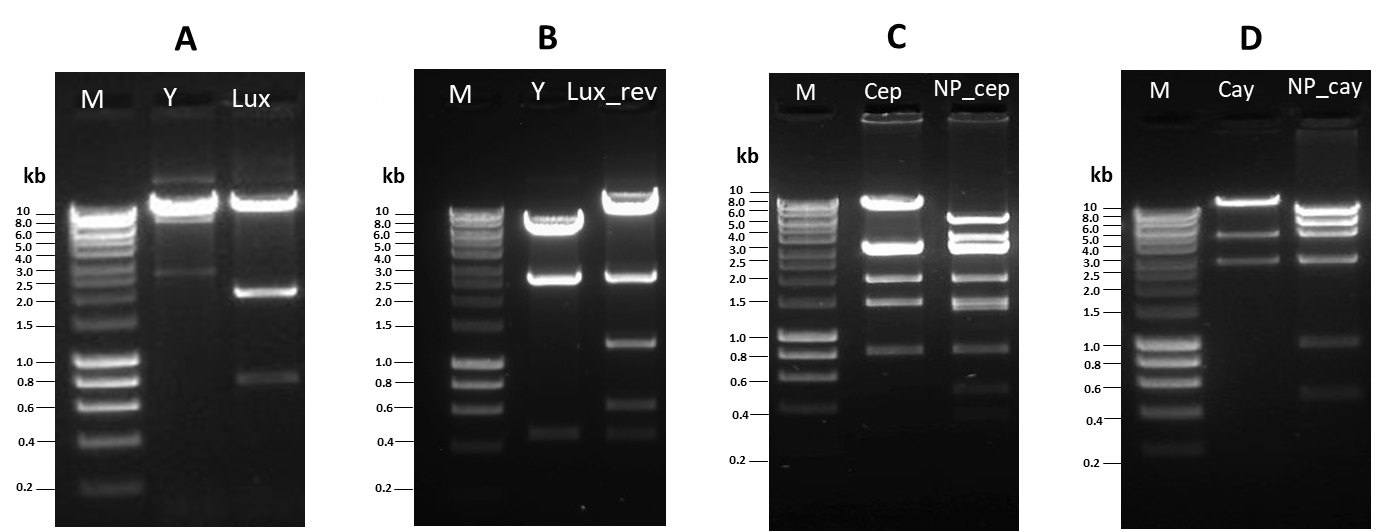


## **Figure S1. Restriction digest gel electrophoresis of the plasmid constructs.**

(A) Restriction digest of pMLBAD_yeast (Y) and pMLBAD_yeast_luxCDABE (Lux) with HindIII and EcoRI with expected fragments of 9530 bp and 50 bp for Y; and 10,582 bp, 1990 bp, 1982 bp and 782 bp for Lux. (B) Restriction digest of pMLBAD_yeast (Y) and pMLBAD_yeast_luxCDABE_rev (Lux_rev) with NcoI and AseI with expected fragments of 6493 bp, 2624 bp, 468 bp for Y; and 10,239 bp, 2671 bp, 1221 bp, 636 bp, 468 bp and 105 bp for Lux_rev. (C) Restriction digest of and pMLBAD_yeast_Pbadcep (Cep) and pMLBAD_yeast_NPcep (NP_cep) with NcoI and EcoRI with expected fragments of 9626 bp, 3368 bp, 3284 bp, 3037 bp, 1925 bp, 1419 bp and 797 bp for Cep; and 6255 bp, 4093 bp, 3368bp, 3284 bp, 3037 bp, 1925 bp, 1419 bp, 1333 bp, 797 bp, 491 bp, 340 bp and 65 bp for NP_cep. (D) Restriction digest of and pMLBAD_yeast_Pbadcay (Cay) and pMLBAD_yeast_NPcay (NP_cay) with NcoI and EcoRI with expected fragments of 15,265 bp, 4722 bp and 2728 bp for Cay; and 8874 bp, 6255 bp, 4722 bp, 2728 bp, 934 bp and 491 bp for NP_cay.


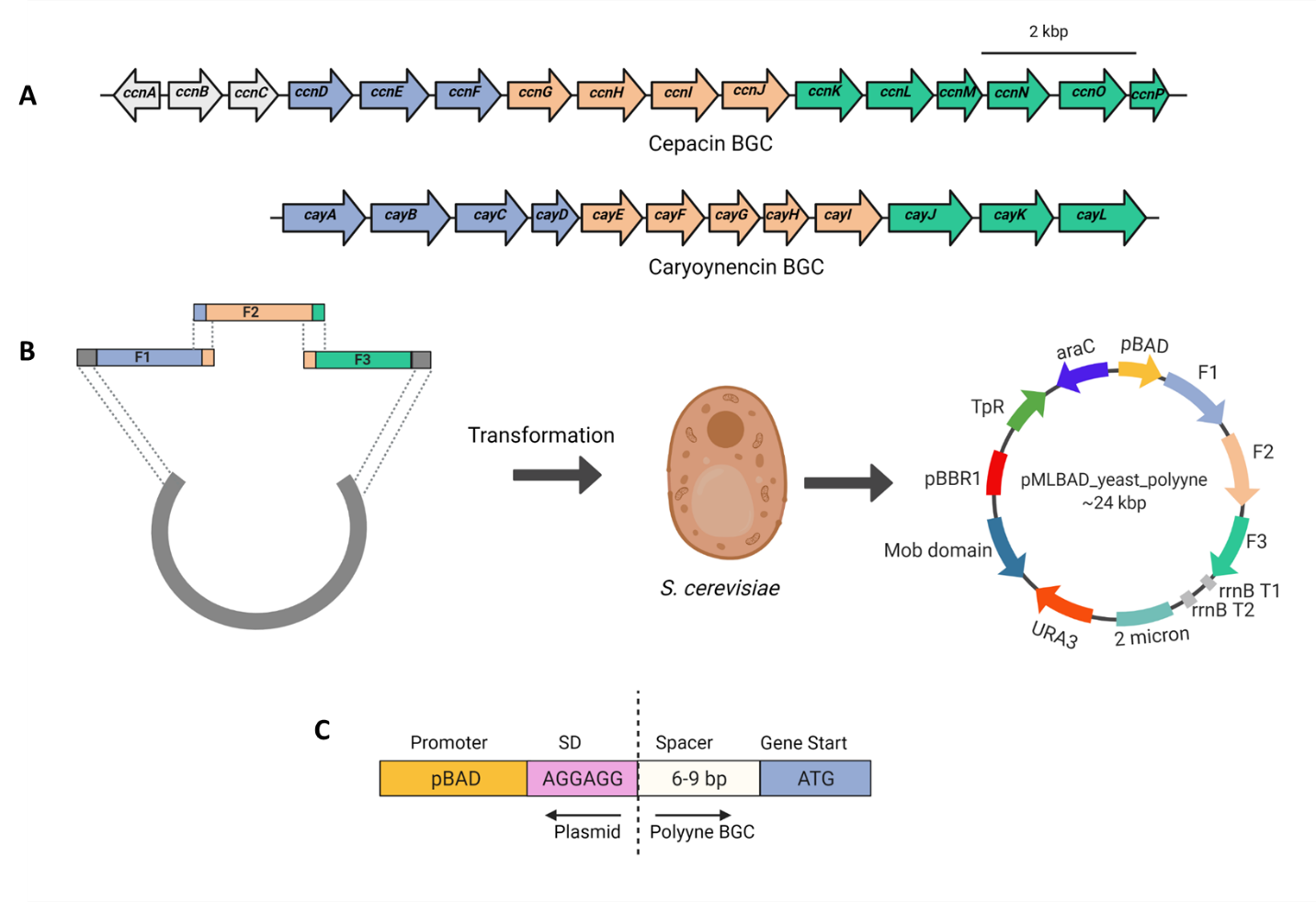


**Figure S2. Cloning of polyyne gene clusters cepacin and caryoynencin in yeast adapted pMLBAD.** (A) The gene arrangement of cepacin BGC (biosynthetic gene cluster) from B. ambifaria BCC0191 and the caryoynencin BGC from B. gladioli BCC1697. (B) The polyyne BGCs were cloned downstream of the Pbad promoter of pMLBAD_yeast in 3 overlapping fragments, F1, F2, and F3 (see Table S5 for PCR primers), by homologous recombination in S. cerevisiae. (C) The cloning strategy employed was such that the first gene of the polyyne BGC, preceded by a native spacer of 9bp (cepacin) or 6 bp (caryoynencin), was placed immediately downstream, of the Shine-Dalgarno (SD) sequence of the pMLBAD vector.


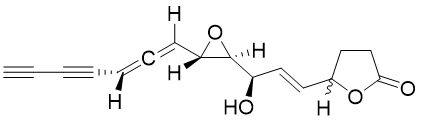


Cepacin A

Chemical formula: C_16_H_14_O_4_

Exact mass: 270.0892

[M+H]^+^: 271.0965

[M+Na]^+^: 293.0784

**A**


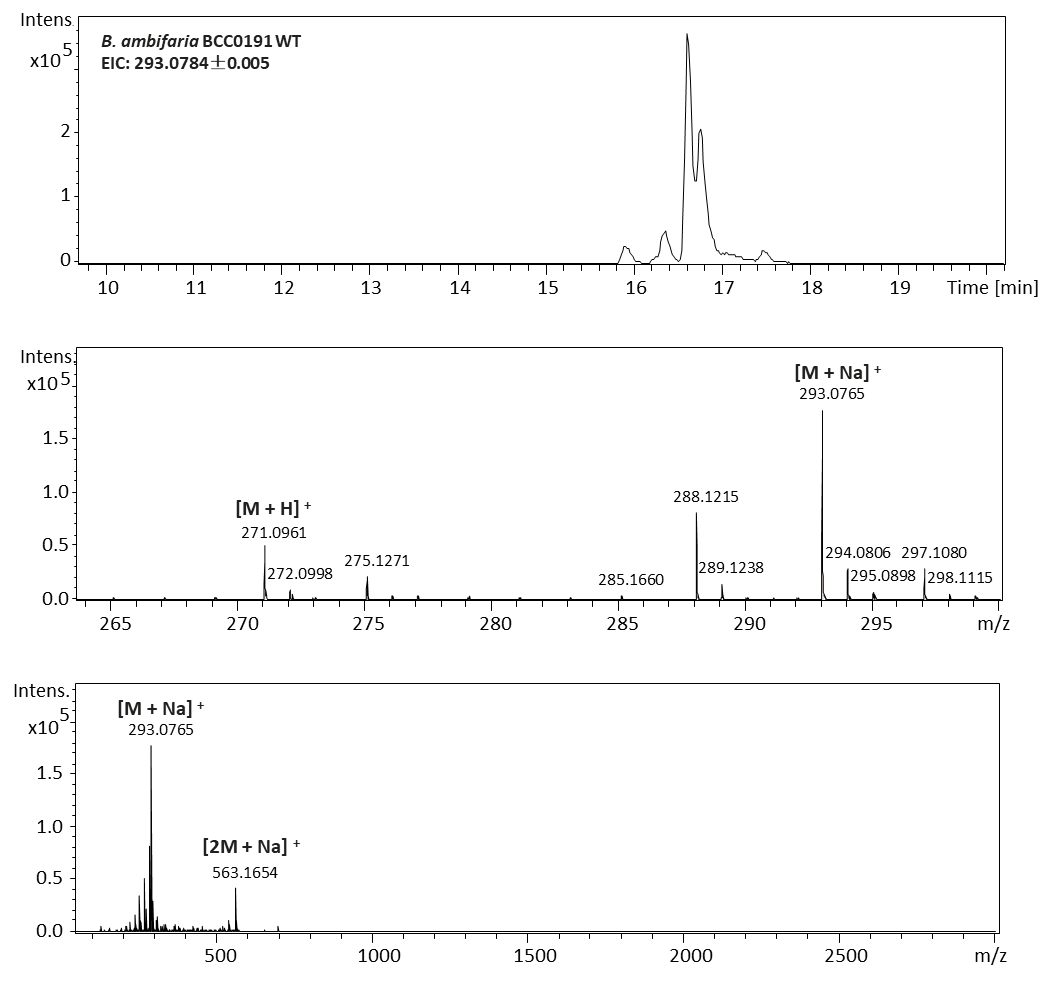


**B**


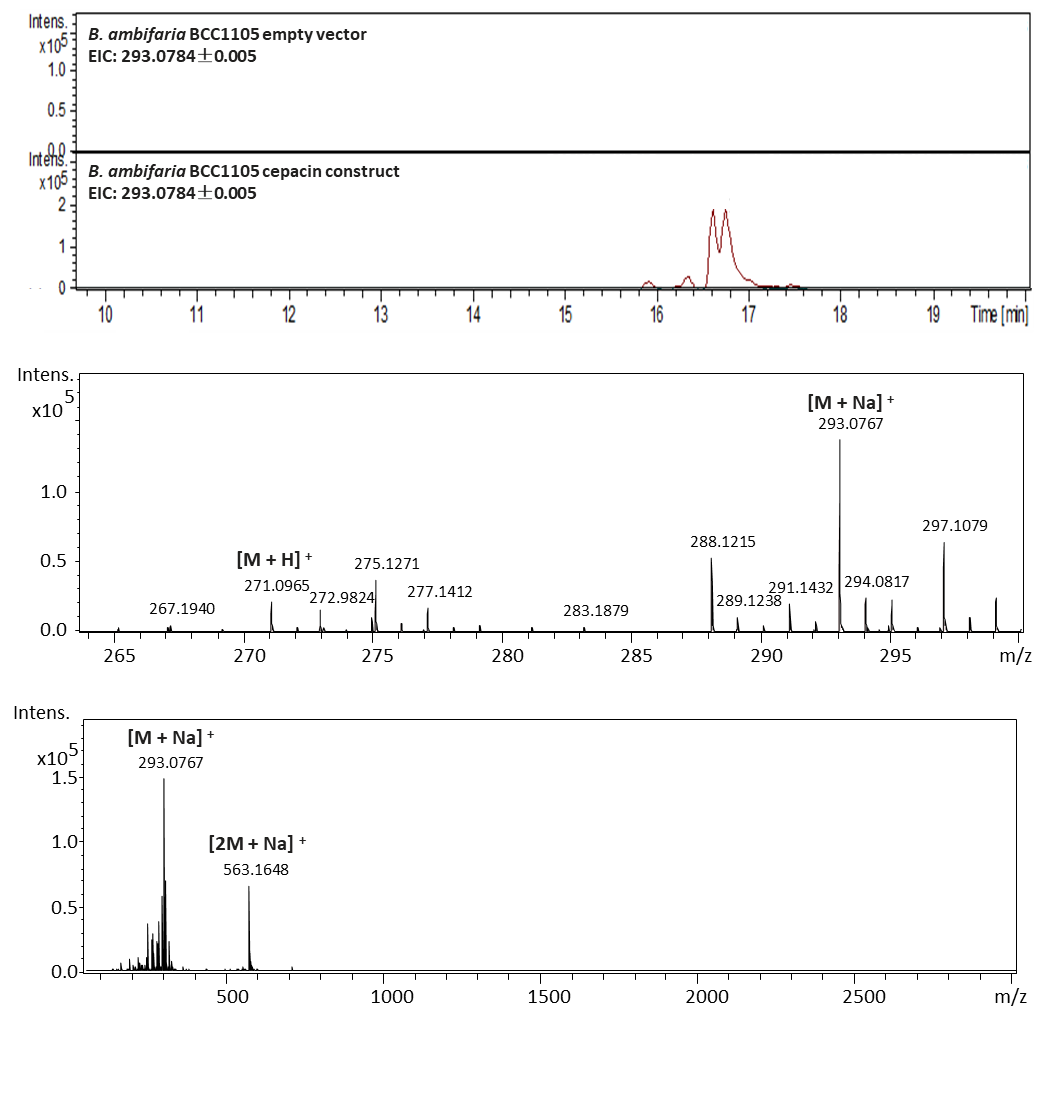


**C**


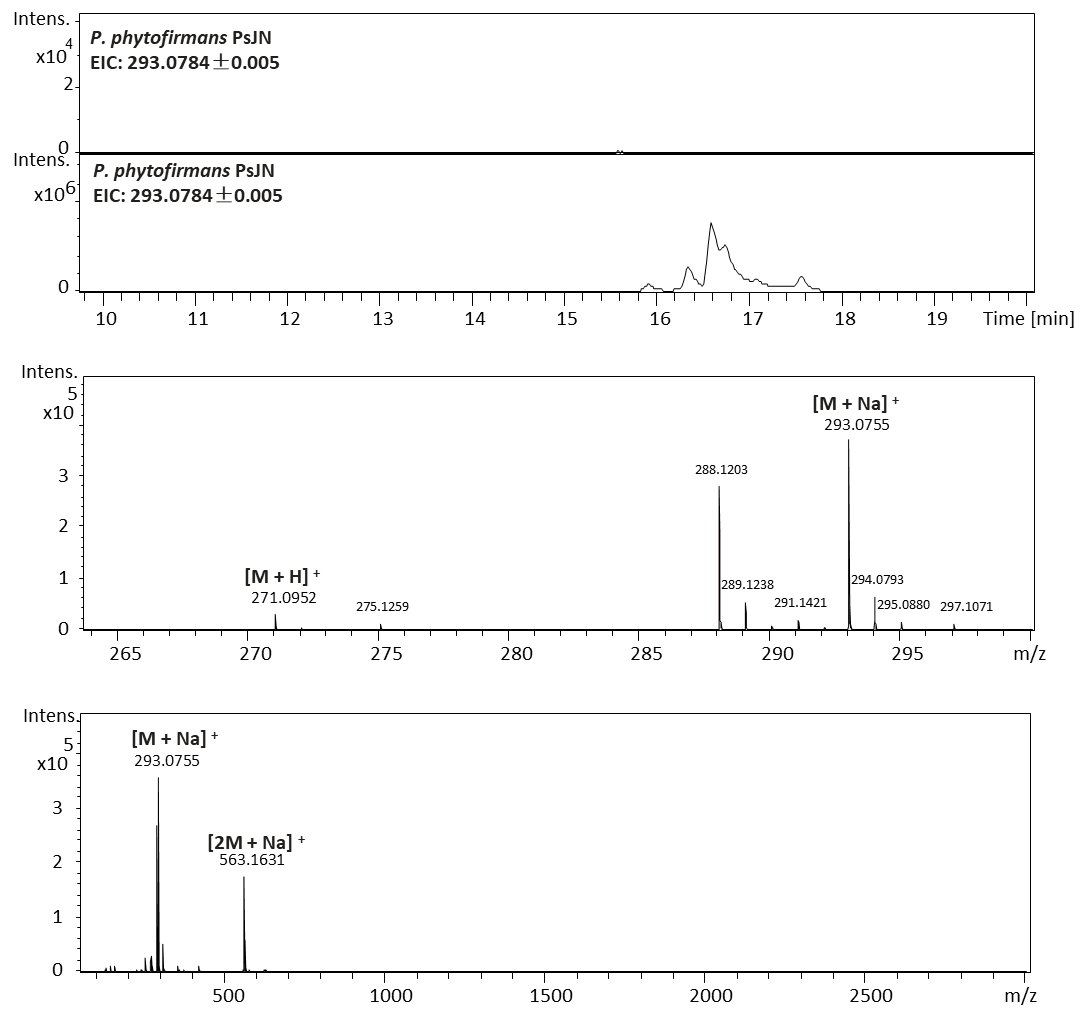


**D**

**Figure S3. Heterologous expression of cepacin by *B. ambifaria* BCC1105 and *P. phytofirmans* PsJN.** Structure of cepacin and calculated masses (A). The extracted ion chromatograms (EICs) for 15.8-17.0 min and high resolution mass spectra are displayed for the major peaks in the chromatograms. Analysis of the following strains is shown: *B. ambifaria* BCC0191 wild type (B) and the cepacin heterologous hosts *B. ambifaria* BCC1105 (C) and *P. phytofirmans* PsJN (D).

**Figure S4. Heterologous expression of caryoynencin by *B. ambifaria* BCC1105, *B. ambifaria* BCC0191 and *P. phytofirmans* PsJN.** Structure of caryoynencin and calculated masses (A). The extracted ion chromatograms (EICs) for 18.5-19.0 min and high resolution mass spectra for the major peaks are shown. Analysis of the following strains is shown in each respective panel: *B. gladioli* BCC1697 wild type (B) and the caryoynencin heterologous hosts *B. ambifaria* BCC1105 (C), *B. ambifaria* BCC0191 (D)**,** *P. phytofirmans* PsJN (E), *P. bannensis* BCC1915 (F), *P. tropica* BCC1950 (G)*, P. sp* BCC1909 (H).

Caryoynencin

Chemical formula: C_18_H_16_O_3_

Exact mass: 280.1099

[M+Na]^+^: 303.0997

[M –H_2_O + H]^+^: 263.1072


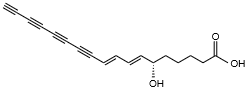


**A**


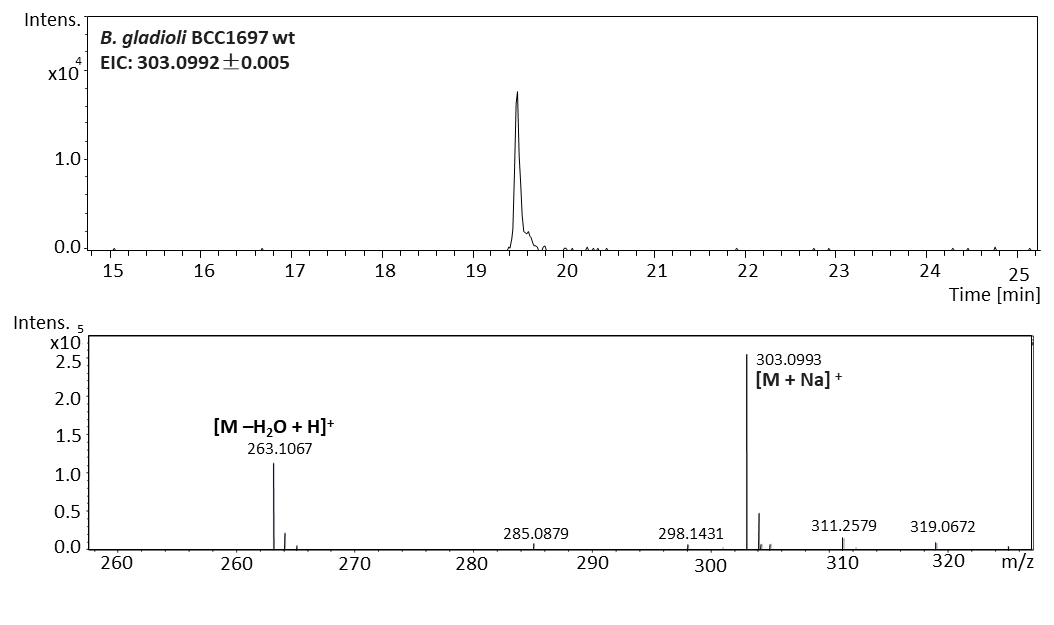


**B**

**C**


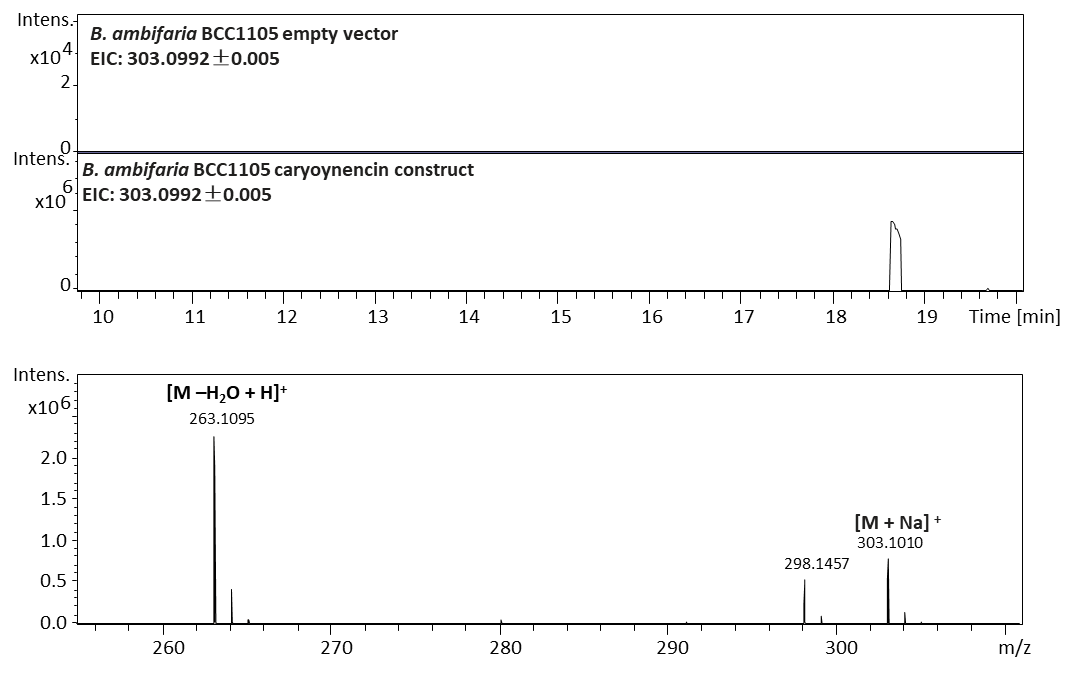

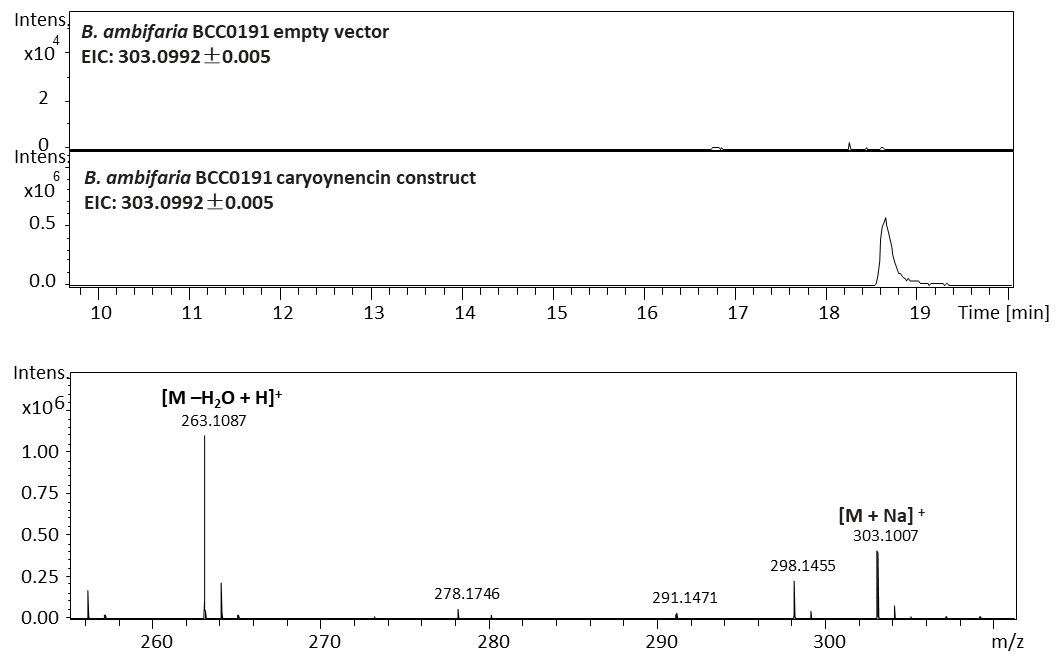


**D**


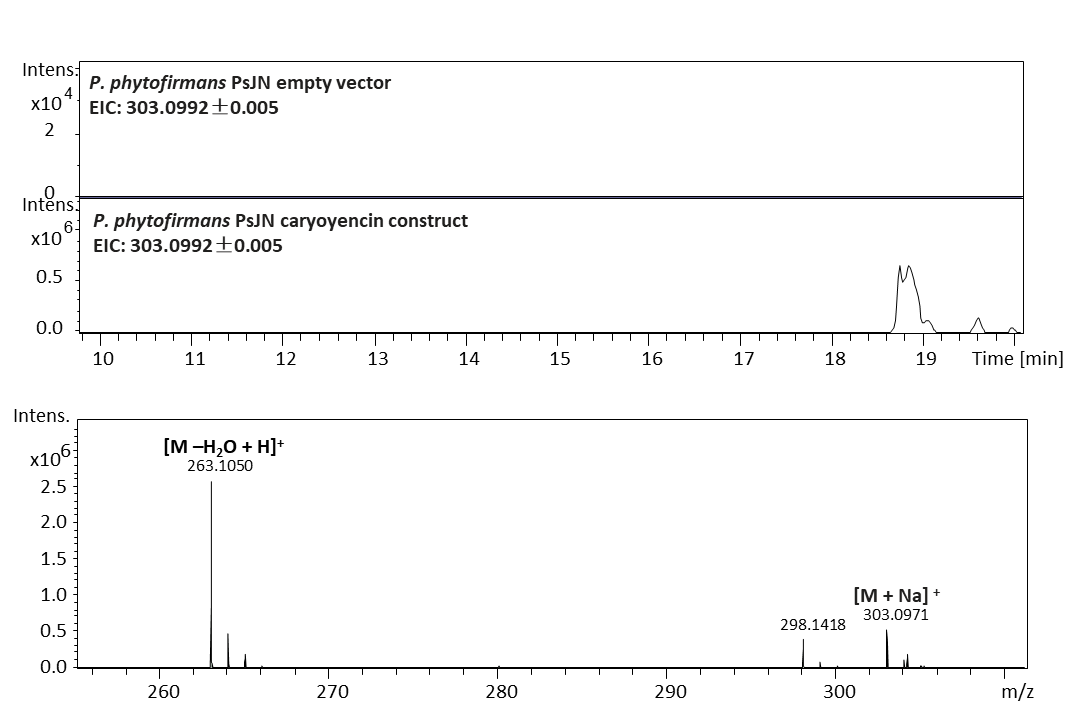


**E**


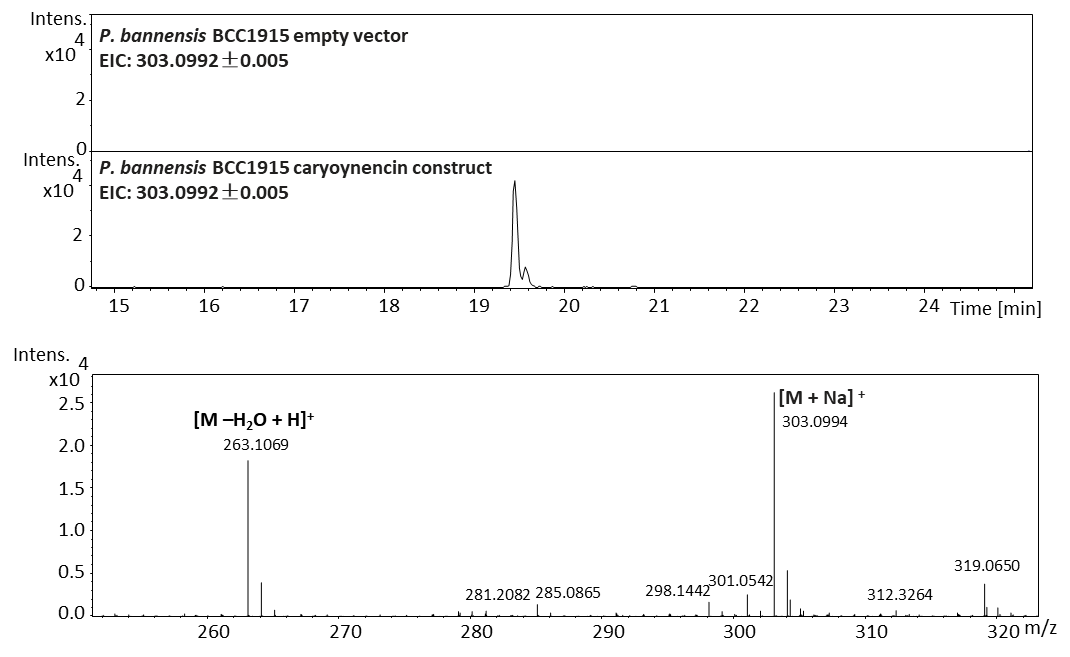


**F**


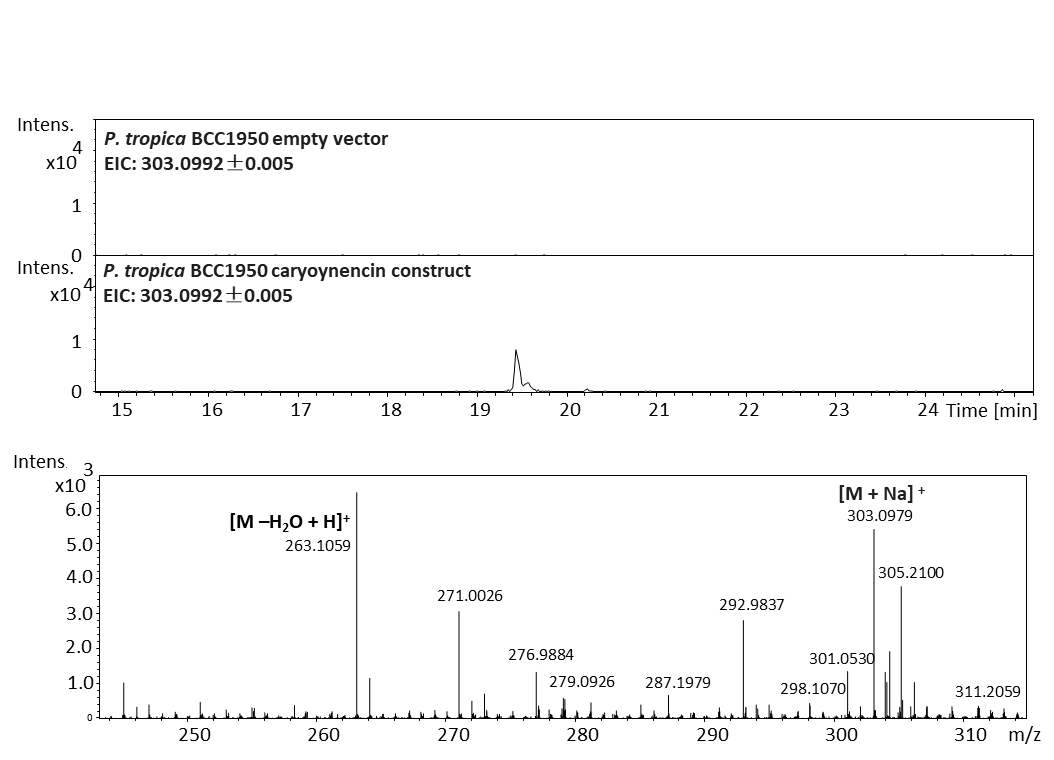


**G**


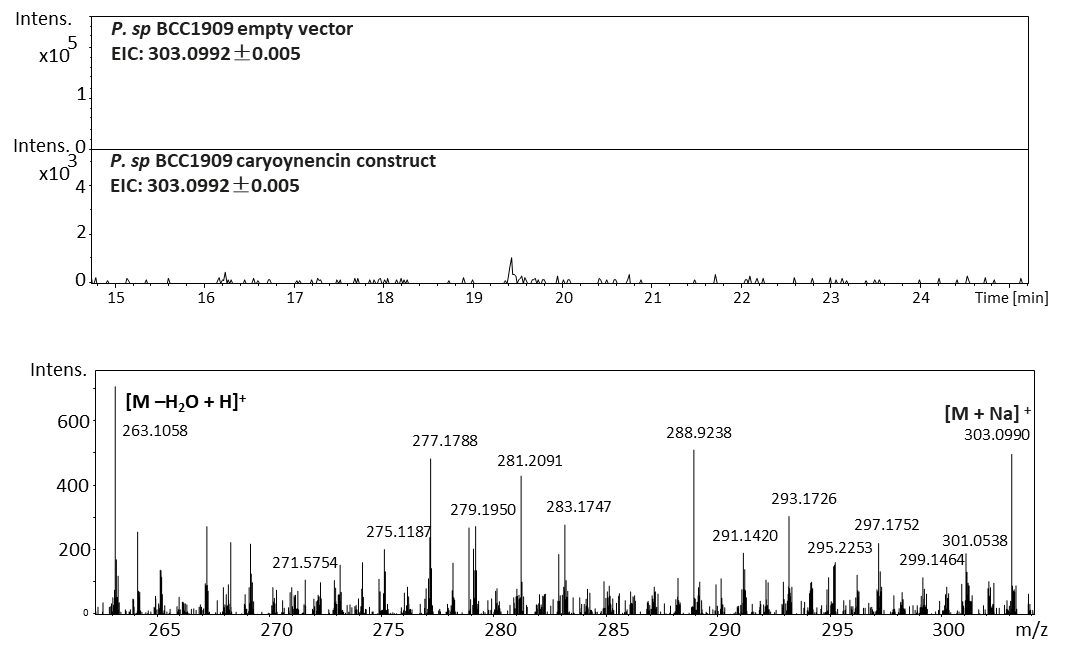


**H**

**Figure S5. pMLBAD constructs copy number and stability of pMLBAD::luxCDABE.** Bacterial hosts containing the pMLBAD::luxCDABE constructs were passaged in BSM-G broth and the relative luminescence units normalized per optical density (A) and the % resistant colonies (B) were recorded at each passage. Each point represents an independent experiment. Simple linear regression was fitted to each data set with 95% CI are represented by the dotted lines. For each data set the regression equation, the R^2^ value and p-value (significance of the regression line from zero) are shown. (C) The copy number, determined by qPCR,for each plasmid construct in each of the heterologous hosts is displayed as the mean value of four independent experiments and the standard deviation is shown in brackets.


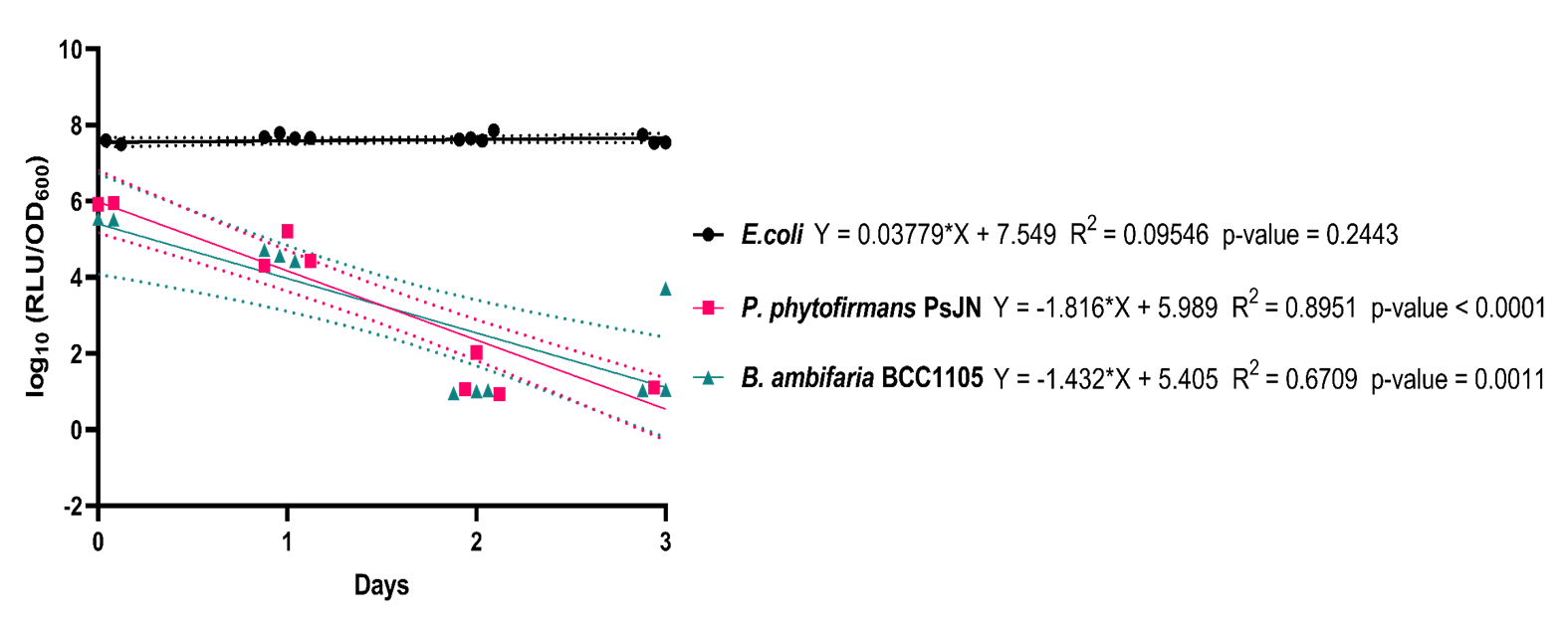

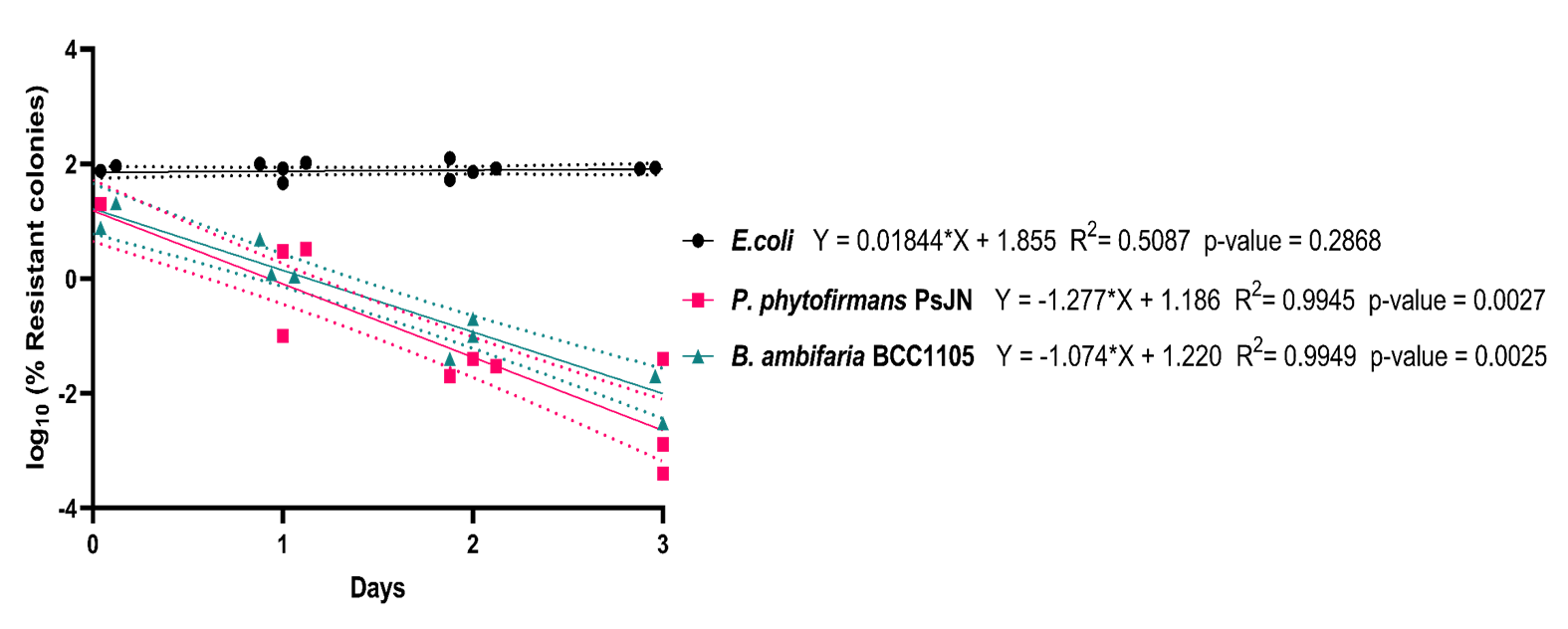

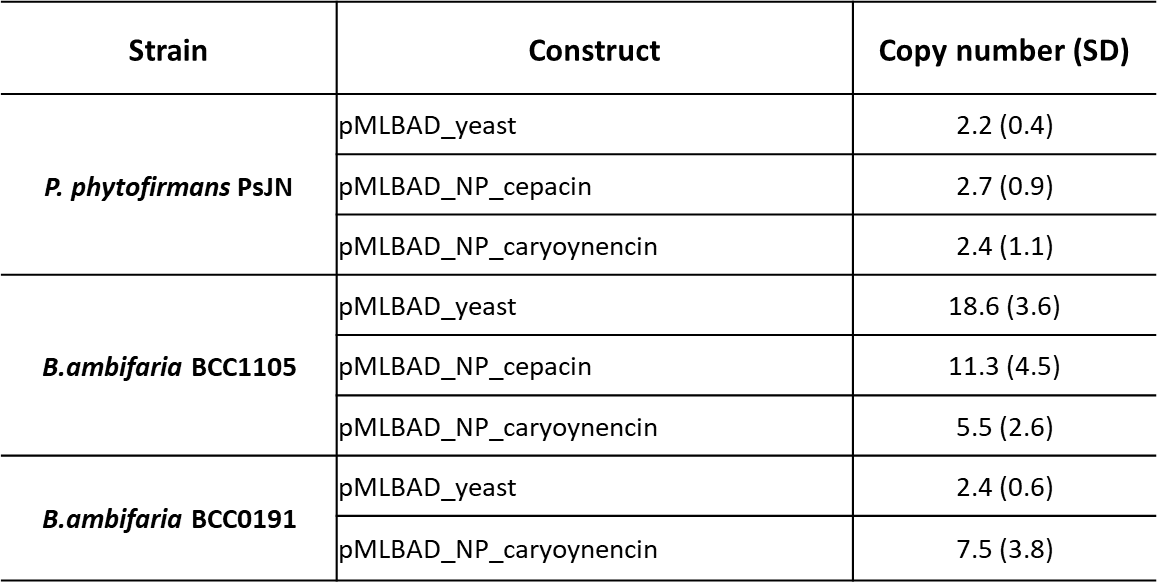


**A**

**B**

**C**

**Figure S6 Temperature differential growth of *Paraburkholderia* panel.** Heatmap summary of the growth parameters of 43 screened *Paraburkholderia* strains at 30°C vs 37°C. Growthcurver R package (Sprouffske, K. and Wagner, A., 2016) was used to calculate the carrying capacity (K) and area under the curve (AUC). The mean value of 3 independent biological replicates for calculated for each growth parameter.

The p-values for K and AUC at 30°C vs 37°C shown were determined by two-way ANOVA (Fishers LSD post-hoc test) in GraphPad Prims 9.3.1.


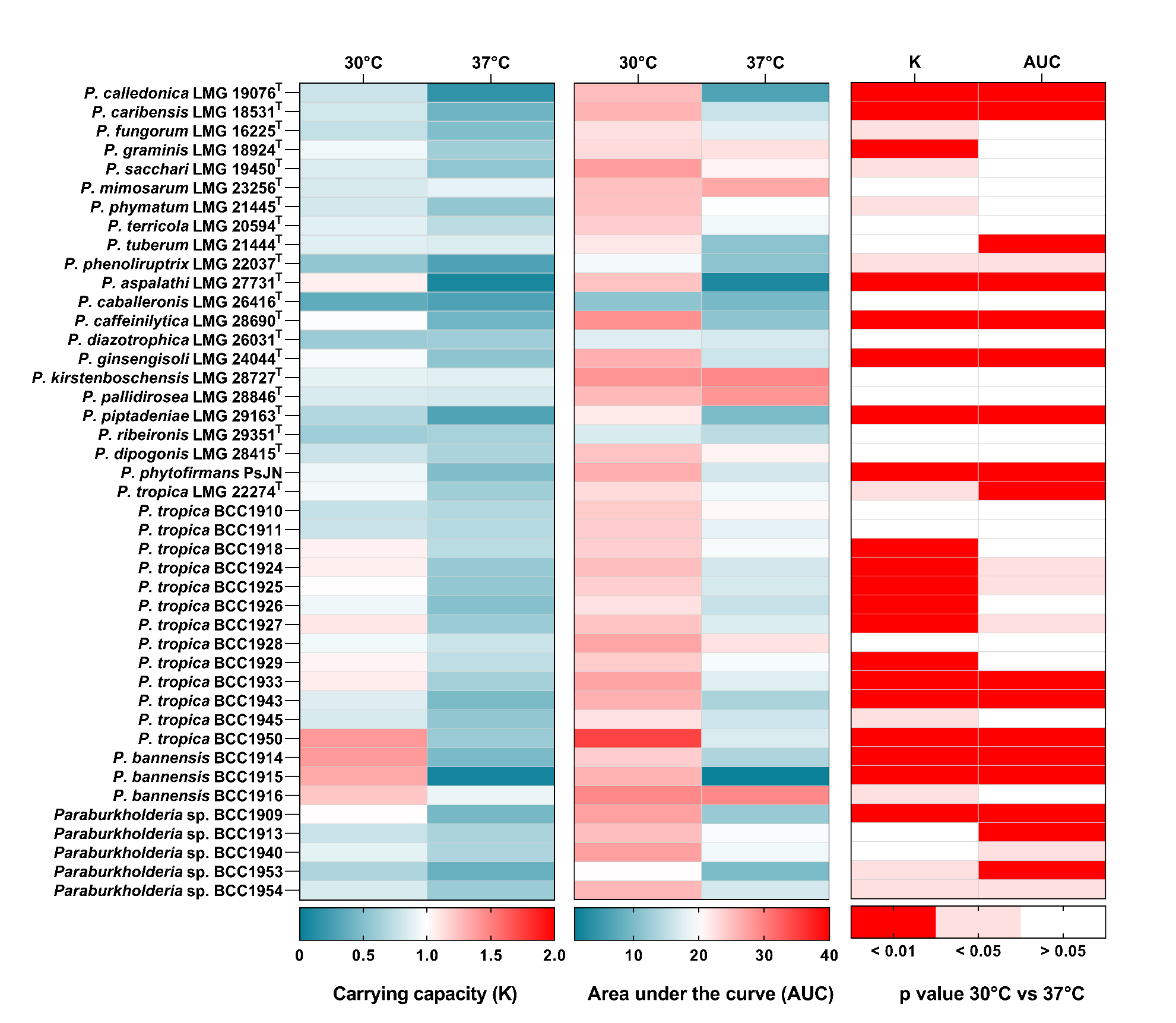


**Figure S7. Temperature differential growth of selected *Paraburkholderia* strains.** Growth curves of *Paraburkholderia* strains with area under the curve and carrying capacity significantly (p < 0.01, two-way ANOVA with Fishers LSD post-hoc test) larger at 30°C than at 37°C. Growth was performed in BSM-G and the optical density measurements were taken every 15 minutes for 48 hours at 420-550nm wavelength using a Bioscreen C instrument (see Supplementary Methods).


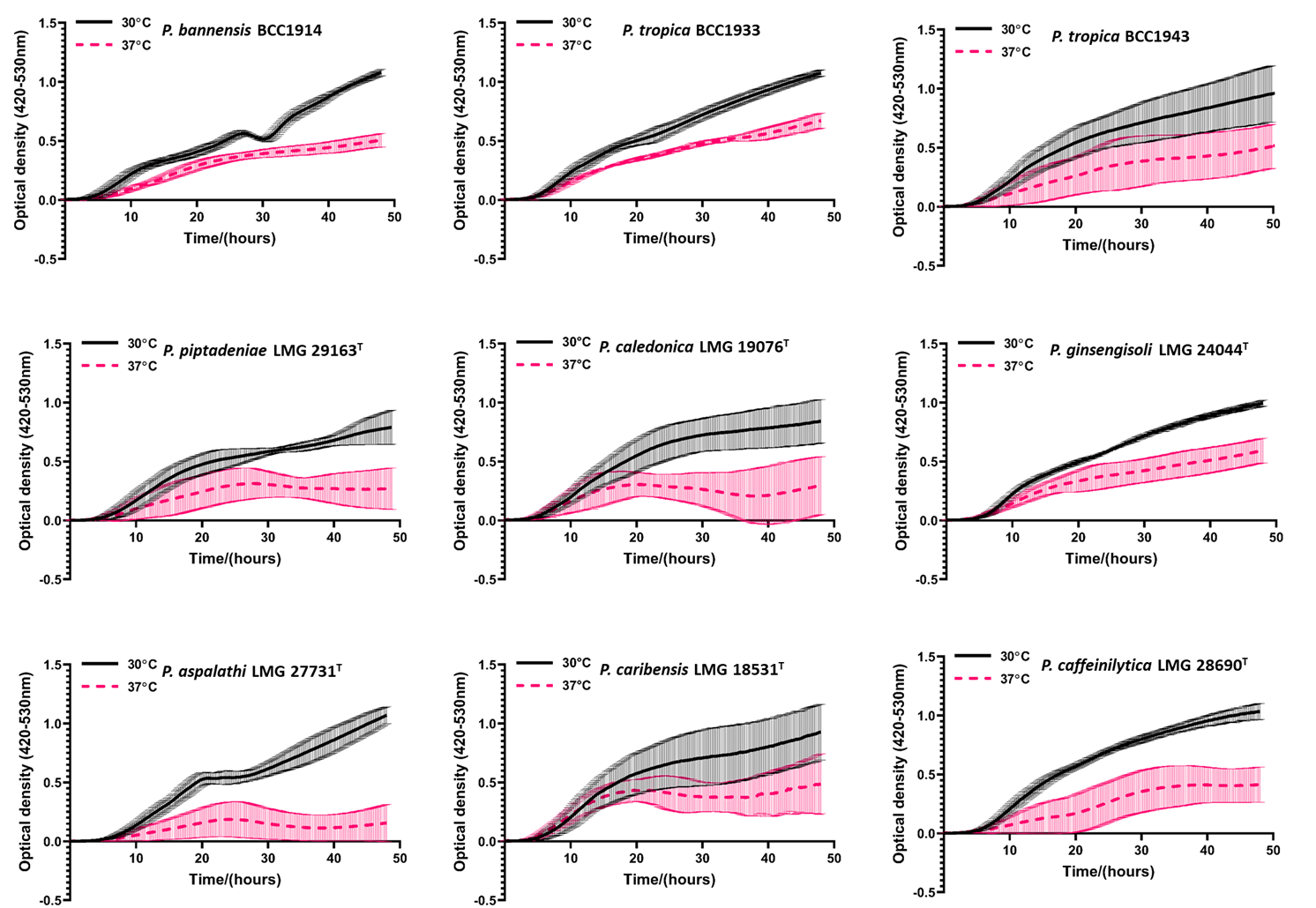


**Figure S8. Bioactivity of recombinant *Paraburkholderia* strains against *Gl. ultimum*.** The bioactivity of the four *Paraburkholderia* strains containing the caryoynencin construct against *Gl. ultimum* determined via contact antagonism assay on BSM-G media.


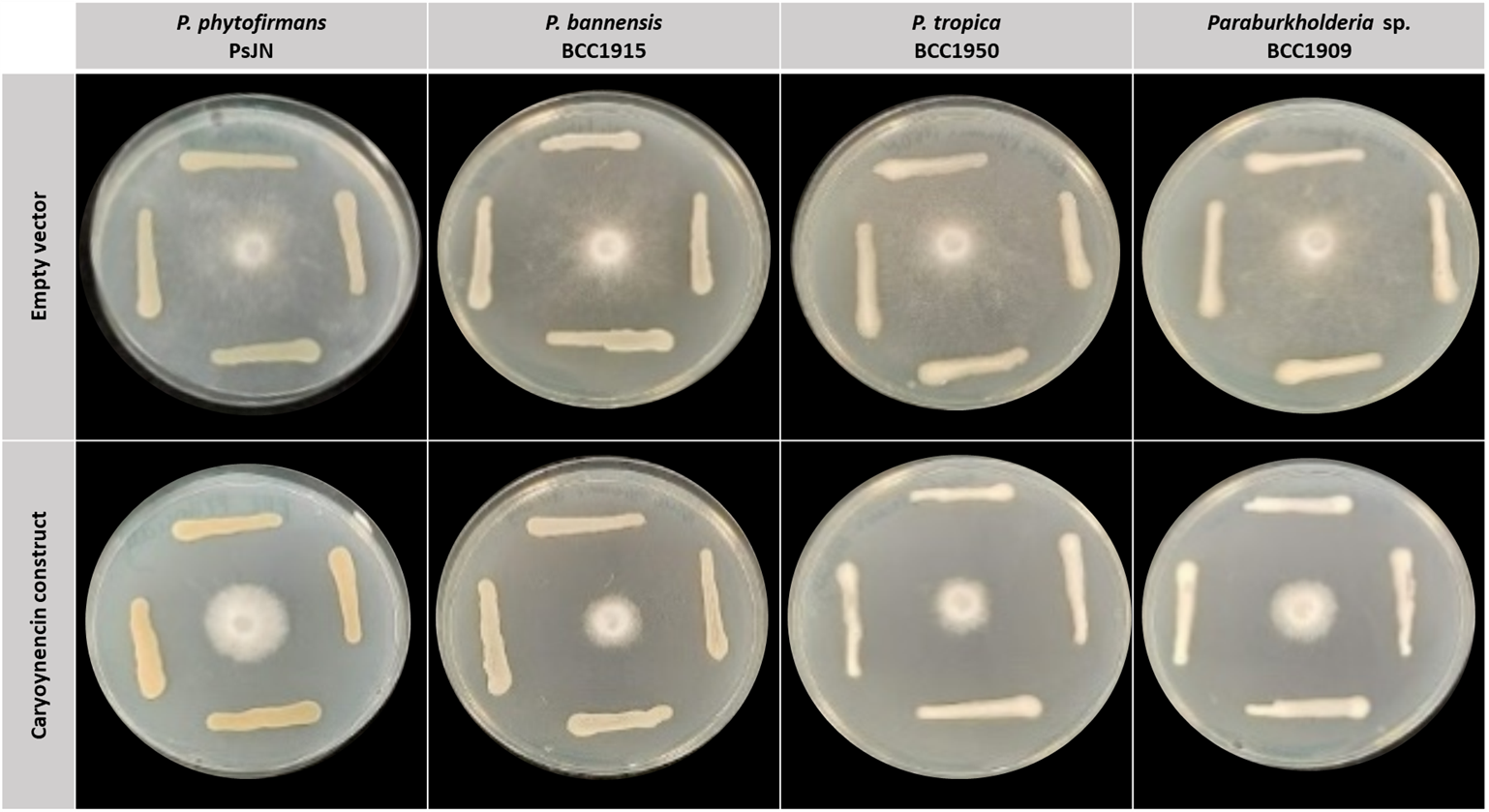


# **References**

Alswat A (2020) The biotechnological potential of natural populations of *Burkholderiales* bacteria for antibiotic production. Thesis, Cardiff University.

Atlas RM (2010) Handbook of microbiological media. CRC Press, Boca Raton, FL.

Craig FF, Coote JG, Parton R, Freer JH & Gilmour NJL (1989) A plasmid which can be transferred between *Escherichia* *coli* and *Pasteurella* *haemolytica* by electroporation and conjugation. J Gen Microbiol 135: 2885-2890.

Dalmastri C, Chiarini L, Cantale C, Bevivino A & Tabacchioni S (1999) Soil type and maize cultivar affect the genetic diversity of maize root-associated *Burkholderia* *cepacia* populations. Microbial Ecol 38: 273-284.

Hareland WA, Crawford RL, Chapman PJ & Dagley S (1975) Metabolic function and properties of 4-hydroxyphenylacetic acid 1-hydroxylase from *Pseudomonas* *acidovorans*. J Bacteriol 121: 272-285.

Datsenko KA & Wanner BL (2000) One-step inactivation of chromosomal genes in *Escherichia* *coli* K-12 using PCR products. Proc Natl Acad Sci USA 97: 6640-6645

Figurski DH & Helinski DR (1979) Replication of an origin-containing derivative of plasmid RK2 dependent on a plasmid function provided in trans. Proc Natl Acad Sci USA 76: 1648-1652

Holden MT, Seth-Smith Hm Fau - Crossman LC, Crossman Lc Fau - Sebaihia M, et al. (2009) The genome of *Burkholderia* *cenocepacia* J2315, an epidemic pathogen of cystic fibrosis patients.

Jones C, Webster G, Mullins AJ, Jenner M, Bull MJ, Dasht Y et al. (2021) Kill and cure: genomic phylogeny and bioactivity of *Burkholderia* *gladioli* bacteria capable of pathogenic and beneficial lifestyles. Microb Gen 7. https://doi.org/10.1099/mgen.0.000515

Lefebre MD & Valvano MA (2002) Construction and evaluation of plasmid vectors optimized for constitutive and regulated gene expression in *Burkholderia* *cepacia* complex isolates. Appl Environ Microbiol 68: 5956-5964

Mahenthiralingam E, Song LJ, Sass A, White J, Wilmot C, Marchbank A, et al. (2011) Enacyloxins are products of an unusual hybrid modular polyketide synthase encoded by a cryptic *Burkholderia* *ambifaria* genomic island. Chem Biol 18: 665-677.

Mao W, Lewis JA, Hebbar PK & Lumsden RD (1997) Seed treatment with a fungal or a bacterial antagonist for reducing corn damping-off caused by species of *Pythium* and *Fusarium*. Plant Dis 81: 450-454

Mullins AJ, Webster G, Kim HJ, et al. (2021) Discovery of the *Pseudomonas* Polyyne Protegencin by a Phylogeny-Guided Study of Polyyne Biosynthetic Gene Cluster Diversity. mBio 12: e00715-00721.

Mullins AJ, Murray JAH, Bull MJ, et al. (2019) Genome mining identifies cepacin as a plant-protective metabolite of the biopesticidal bacterium *Burkholderia* *ambifaria*. Nat Microbiol 4: 996-1005

Ogier J-C, Pagès S, Galan M, Barret M & Gaudriault S (2019) *rpoB*, a promising marker for analyzing the diversity of bacterial communities by amplicon sequencing. BMC Microbiol 19: 171.

Pahirulzaman KAK, Williams K & Lazarus CM (2012) A toolkit for heterologous expression of metabolic pathways in Aspergillus oryzae. Natural Product Biosynthesis by Microorganisms and Plants, Pt C 517: 241-260.

Rushton L, Sass A, Baldwin A, Dowson CG, Donoghue D & Mahenthiralingam E (2013) Key role for efflux in the preservative susceptibility and adaptive resistance of *Burkholderia* *cepacia* complex bacteria. Antimicrob Agents Chemother 57: 2972-2980.

Sass AM, Schmerk C, Agnoli K, Norville PJ, Eberl L, Valvano MA & Mahenthiralingam E (2013) The unexpected discovery of a novel low-oxygen-activated locus for the anoxic persistence of *Burkholderia* *cenocepacia*. ISME J 7: 1568-1581.

Webster G, Parkes RJ, Fry John C & Weightman Andrew J (2004) Widespread Occurrence of a Novel Division of Bacteria Identified by 16S rRNA Gene Sequences Originally Found in Deep Marine Sediments. Appl Environ Microbiol 70: 5708-5713

Winson MK, Swift S, Hill PJ, Sims CM, Griesmayr G, Bycroft BW, Williams P & Stewart G (1998) Engineering the *luxCDABE* genes from *Photorhabdus* *luminescens* to provide a bioluminescent reporter for constitutive and promoter probe plasmids and mini-Tn5 constructs. FEMS Microbiol Lett 163: 193-202
